# Coevolved Canonical Loops Conformations of Single-Domain Antibodies: A Tale of Three Pockets Playing Musical Chairs

**DOI:** 10.3389/fimmu.2022.884132

**Published:** 2022-06-03

**Authors:** Francis Gaudreault, Christopher R. Corbeil, Enrico O. Purisima, Traian Sulea

**Affiliations:** Human Health Therapeutics, National Research Council Canada, Montreal, QC, Canada

**Keywords:** single-domain antibody, CDR prediction, antibody design, canonical structure, structural determinants, sequence preferences, canonical pairing, CDR evolution

## Abstract

Single-domain antibodies (sdAbs) are a promising class of biotherapeutics with unique structural traits within their paratope region. The distribution of canonical conformations explored by their complementarity determining region (CDR) loops differs to some extent from conventional two-chain Fv fragments of monoclonal antibodies (mAbs). In this study, we explored in detail the canonical structures of sdAb CDR-H1 and CDR-H2 loops and compared those with mAbs from the IGHV3 and IGHV1 gene families. We surveyed the antibody structures catalogued in SAbDab and clustered the CDR canonical loops in Cartesian space. While most of the sdAb clusters were sub-populations of previously defined canonical Fv conformations of CDR-H1 and CDR-H2, our stricter clustering approach defined narrower clusters in sequence-space. Meticulous visual inspection of sub-populations allowed a clearer understanding of sequence-structure relationships. The packing densities within structural pockets contacted by CDR-H1 and CDR-H2 canonical conformations were analyzed on the premise that these pockets cannot be left vacant as they would leave exposed supportive hydrophobic residues. The fine resolution of the canonical clusters defined here revealed unique signatures within these pockets, including distinct structural complementarities between CDR-H1 and CDR-H2 canonical clusters, which could not be perceived with the previous coarser clusters. We highlight examples where a single residue change in CDR-H1 sequence is sufficient to induce a dramatic population shift in CDR-H2 conformation. This suggests that preferences in combining CDR-H1 and CDR-H2 emerged naturally during antibody evolution, leading to preferred sets of conserved amino acids at key positions in the framework as well as within the CDR loops. We outline a game of musical chairs that is necessary to maintain the integrity of the antibody structures that arose during evolution. Our study also provides refined CDR-H1 and CDR-H2 structural templates for sdAb homology modeling that could be leveraged for improved antibody design.

## Introduction

The variable regions of heavy-chain antibodies (HCAbs), which are devoid of a light chain, are commonly referred to as single-domain antibodies (sdAbs; V_H_H or nanobody) as opposed to conventional two-chain Fv fragments of monoclonal antibodies (mAbs) which include both heavy-chain (VH) and light-chain (VL) variable domains. The sdAbs have many desirable properties that make them promising biotherapeutics, such as high antigen binding affinity, thermostability, solubility and tissue penetration, as well as low production costs (1, 2). Nonetheless, the full potential of these molecules has yet to be uncovered, with a single nanobody approved for clinical use at the present time (3). To this practical end, an elevated understanding of sdAb structural diversity can aid further optimization efforts towards their engineering and provide an alternative and complementary format for biologics design.

The Protein Data Bank (PDB) is a rich source of structural data of antibodies that can be leveraged to help in this endeavor. However, there is an inherent representation bias in the PDB towards antibodies from the IGHV3 and IGHV1 families of germline genes (from human and mouse) as opposed to a relatively low availability of sdAb structures. Numerous studies leveraged the PDB to highlight some distinct properties of the sdAb paratopes that differentiate them from conventional mAbs. Lacking the VL domain, their complementarity-determining region (CDR) is composed of only two canonical loops (CDR-H1 and CDR-H2) and one hypervariable loop (CDR-H3). The sdAbs can bury as much surface area as the Fv fragments of mAbs upon antigen binding but with a higher density of contacts and interactions (4). To compensate for their smaller paratope, in many instances, antigen binding to sdAbs involves contributions from residues outside the CDR (5). To the same end, their CDR-H3 loop tends to be on average longer and more protuberant thus allowing them to bind more concave epitopes. Therefore, sdAbs appear to target a set of proteins that is complementary to those targeted by conventional mAbs (6). It was also observed that their distribution of canonical structures falls outside the standard classification (4) in part due to increased sequence diversity within their CDRs (7). More sequence-structure relationship understanding of sdAbs can prove useful to their design and optimization.

sdAbs were first discovered to be naturally occurring in dromedary camels but were later shown to also have origins in other Camelidae such as llama and alpaca (8). The sequences of publicly accessible sdAb structures in the PDB have their origin from the IGHV (VH) or IGHVH (V_H_H) gene families (9, 10). The two families evolved as independent machineries for immunity in Camelidae from the many constraints imposed during evolution and gave rise to unique sequence conservation profiles (11, 12). For instance, the IGHV gene family shows distinct sequence preferences in its framework and CDR when compared to the IGHVH family (10, 13). Camelidae antibodies provide a robust therapeutic antibody platform given their strong sequence homology to human monoclonal antibodies from the IGHV3 family and from the IGHV1 family to a lesser extent (14). This has prompted the development of autonomous human VH domain antibodies (dAbs) for therapeutics use (15), which have seen a noticeable increase in number in the PDB. Of note are the cases of primitive antibodies present in cartilaginous fish (V_NAR_) for which only a handful of structures are accessible in the PDB. Notwithstanding their therapeutic potential (16, 17), V_NAR_ antibodies are more difficulty compared to sdAbs or mAbs provided their important sequence divergence and their framework structure essentially lacking the CDR-H2 loop. The conservation of key amino acids is an essential feature to consider in structure prediction as a heavily conserved amino acid is normally indicative of an underlying structural function (18). In fact, a residue change at a conserved position is likely to have a detrimental effect (19) and can propagate a series of structural changes that can affect thermal stability and even correct folding. For antibodies, this could also result in deficiencies in antigen binding. Structurally, the conserved residues can for instance act as strong determinants for defining and supporting canonical structures. Given the unique sequence conservation profiles across various species as well as antibody classes and families, a better understanding of the structural determinants may shed some light into why sdAbs differ in their distribution of canonical structures from the conventional mAbs.

Previous studies highlighted the importance of buried residues in close proximity to the CDR to greatly influence the set of canonical CDR loop conformations. A classic example is the Arg residue at position 71, located in a rigid portion of the antibody framework (20), which was highlighted many years ago as being structurally important (21). Arg-71 structurally supports CDR-H2 by filling a pocket underneath it. The authors hinted that Arg-71 mutation to a smaller residue would trigger a different canonical conformation of CDR-H2 that does not require its structural support. This hypothesis was later confirmed by another study where having a smaller residue at position 71 is typically predictive of the canonical structure H2-10-1 while Arg-71 tends to favour the H2-10-2 canonical conformation (22). Naturally, the level of conservation of such support residues results from their tight packing and physico-chemical complementarity with residues from the canonical CDR loop structures. Hence, computational design of antibodies must involve a tight core packing (23–25) and avoid leaving hydrophobic crevices exposed to solvent between the framework and CDR loops (26, 27). The prediction of the hypervariable CDR-H3 conformation, the longer and most difficult CDR loop to predict, is also easier when provided with a more accurate structural representation of the framework and the canonical CDR loops (28, 29). Overall, more success could be achieved if one could predict with more confidence the foundational structure of the canonical structures along with its support residues.

The classification from North et al. is the most extensive study to-date for predicting the structure of the canonical loops of the CDR (22). This significant work modernized the previous foundational study on the analysis of canonical structures (30–32). The North et al. classification was carried out in light of the exponential increase in size of the PDB allowing the authors to only retain structures of high-quality. The classification is updated periodically with newly deposited antibody structures from the PDB based on a dedicated database called PyIgClassify (33). In the original classification, the CDR-H1 and CDR-H2 loops could be clustered into 12 and 9 canonical structures for the dominant lengths of 13 and 10 amino-acid residues, respectively. The H1-13-1 cluster was observed roughly in 75% of available structures, and the H2-10-1 and H2-10-2 clusters in 55% and 25% of available structures, respectively. However, little is known about the structural determinants that are required to fold a CDR loop sequence into a particular canonical conformation.

The CDR canonical classification of North et al. provided sequence logos for each canonical structure that could be used to extract some meaningful sequence-structure relationships. However, those are not easily interpretable for two reasons. First, the underlying dataset pooled together many classes of antibodies each differing quite significantly in their framework sequence. In fact, the sequence logos are averages from an inherently biased representation of antibodies in the PDB, *i.e.*, certain antibody classes dominate the dataset. With our focus in this study on sdAbs, some sequence-structure relationships specific to sdAbs may have been lost in the previous classification due to amalgamation of antibody classes, making it difficult to assess the transferability of canonical structures between sdAbs and other antibody classes and extract meaningful structural determinants for sdAbs. Secondly, previous clustering criteria were quite permissive and hence do not allow subtle but critical conformational changes to be perceived. The canonical clusters of North et al. span a wide umbrella of conformations that encompass fairly large structural movements and indirectly allow for a certain level of sequence variability. Hence, some important sub-populations of canonical clusters may have been missed.

In this study, we build upon the reference work of North et al. and focus more deeply into CDR-H1 and CDR-H2 canonical structures of sdAbs specifically vis-à-vis those of conventional two-chain antibodies. Our analysis provides a crisper rationalization of sequence-structure relationships that eventually reveals co-evolved structural patterns in terms of mutual structural compatibilities and co-occurrences, as well as a strong dependence on the structure of the underlying framework residues. The outcome of this analysis may also provide updated homology modeling templates for improved antibody design and optimization.

## Methods

Antibody structures were collected from the Structural Antibody Database (SAbDab) database on the release of October 13, 2021 using as search criteria the antibody type V_H_H for the set of single-domain antibodies and the antibody type Fv for the sets of conventional IGHV1 and IGHV3 antibodies (34). A single entry was retained for every unique heavy-chain canonical CDR sequence (CDR-H1–CDR-H2) while giving priority to the structure with the highest resolution. The Chothia numbering scheme was used throughout the study (31). The use of Chothia was a necessary requirement to stay within the same boundaries and remain consistent with a previous study (22). Correspondence in the IMGT Collier de Perles numbering scheme was provided for reference to standards of immunoinformatics (35). The set of residues that compose the CDR-H1, CDR-H2 and CDR-H3 were defined as positions 23 to 35, 50 to 58, and 93 to 102, respectively (IMGT positions 24 to 40, 55 to 66 and 105 to 117). Other regions of interest were defined as follows: the N-terminal region (NT) as positions 1 to 6, and the FR3 region as positions 70 to 78 (IMGT positions 79 to 87). The CDR loop lengths for each entry were determined following a multiple sequence alignment using ClustalW2 (36). Given their better statistics, only entries with the dominant CDR configuration 13-10 were retained, *i.e.*, entries with lengths 13 and 10 amino-acid residues (IMGT lengths 8 and 8) for CDR-H1 and CDR-H2, respectively.

Similar to a previous study that analyzed the CDR region of antibody structure (37), a set of clean structures was built that excludes structures with resolution lower than 3 Å or with any missing backbone atoms in the canonical CDR loops. For the set of sdAb structures where data is the most limited and where more diversity in canonical structures is observed, the removal of structures with missing backbone atoms had negligible impact with only 12 structures being excluded. On the other hand, the removal of poorly resolved structures had a larger impact with 54 structures not included. Out of those 54 structures, 20% and 31% belong to existing major clusters of CDR-H1 and CDR-H2, respectively. The remaining excluded entries were singletons of new minor clusters or populated existing ones but not in sufficient numbers to create new major clusters. The clean structures were clustered on the basis of Cartesian coordinates using the set of backbone atoms (N, Cα, C and O) of the CDR-H1 or the CDR-H2. For clarity, throughout the article the term *canonical* is strictly used when referring to the North et al. clusters, while the term *cluster*, when used in isolation, refers to clusters defined in the current study. A hierarchical clustering based on the ward.D2 linkage method that minimizes variance was performed in R (R Core Team, 2020). The dissimilarity matrix was obtained from the best-fit RMSD following a SVD superposition based on the Kabsch algorithm (38). The clusters were delimited based on an empirical RMSD height cutoff of 1.5 Å. Only clusters with at least three representatives were retained. The centroid of a cluster is defined as the structure closest to an averaged structure of the cluster representatives. The sequence logos of the clusters were generated using the WebLogo standalone software from weblogo.berkeley.edu.

The sausage view used to display the CDR atom fluctuations among representatives of a topological variant (or cluster) implies the calculations of B-factors. The B-factors of the atoms were calculated using the established formula 8/3π^2^ ✕ RMSF^2^, where RMSF are the RMS fluctuations to the centroid structure. The B-factors were weighted down by a factor 4 for visual purposes. The visible Cβ atoms on the sausage view are the ones of the centroid structure. The graphical representations were produced in PyMOL (PyMOL Molecular Graphics System, Version 2.0 Schrödinger, LLC).

Volumes of the regions of interest (V_R_) were built from the set of heavy atoms composing these regions using a standard set of atom radii (39). The structures were superimposed onto the template structure 3k74[B] from which the volumes of the pockets (V_P_) were defined. The regions of interest were excluded for the superposition. The occupancy was defined as the intersection of V_R_ ∩ V_P_ and was expressed in percentage by normalizing to V_P_. The volumes were calculated using the volume calculator from the NRGsuite (40).

## Results

### Distribution of Prevalent CDR Configurations

**Figure 1:**
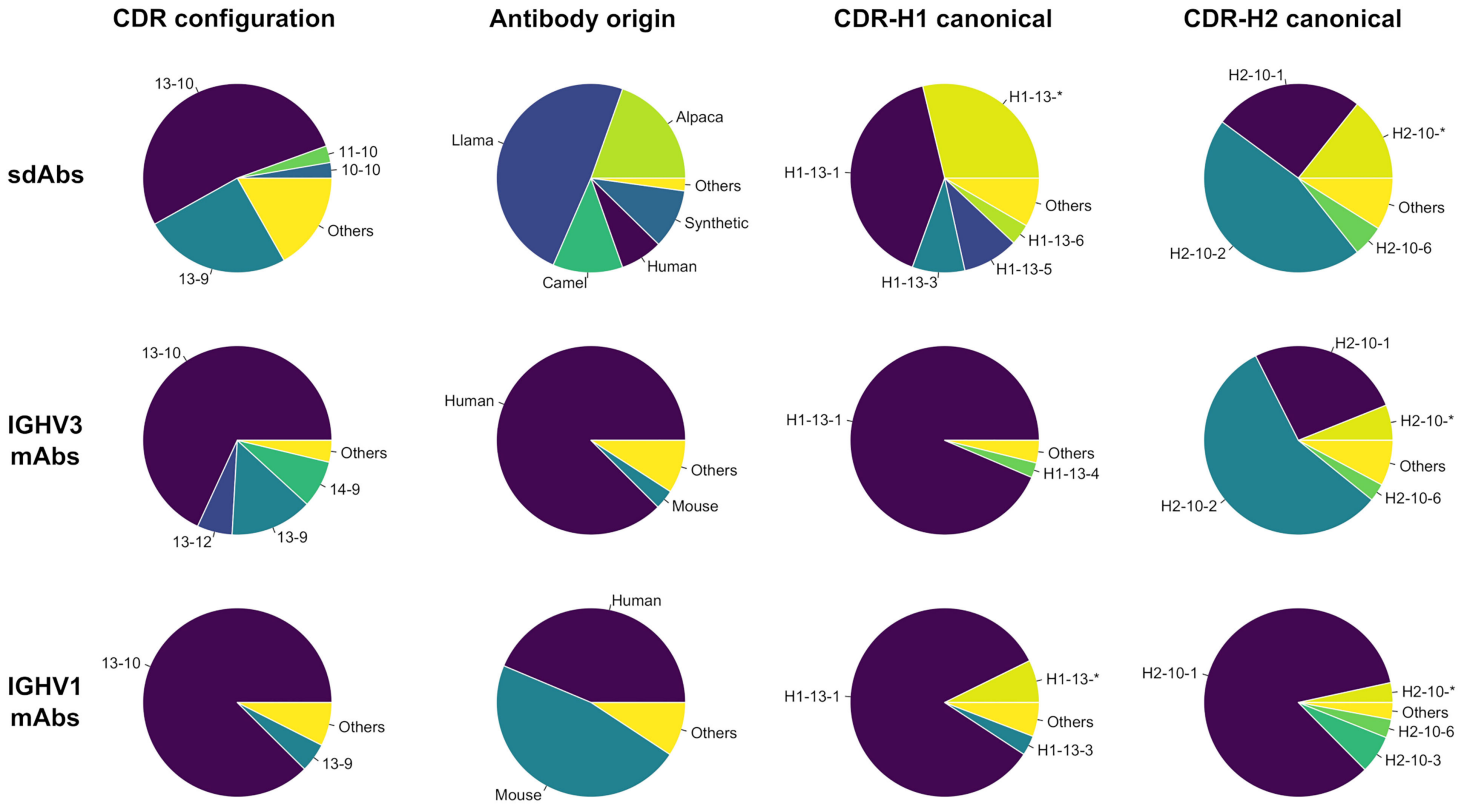
Distributions for the entire set (first column) and set of antibody structures with CDR configuration 13-10 (second, third and fourth columns) are shown for single-domain antibodies and conventional antibodies from the IGHV3 and IGHV1 gene families. The distributions show the groups for (i) CDR configurations with the first and second numbers being the respective lengths for the canonical loops CDR-H1 and CDR-H2; (ii) origins from Human (*Homo sapiens*), Mouse (*Mus musculus*), Llama (*Lama glama*), Camel (*Camelus dromedarius* or *Camelus bactrianus*), Alpaca (*Vicugna pacos*) and Synthetic; and (iii) canonical structures for CDR-H1 and CDR-H2 as reported by PyIgClassify. The groups encompassing less than 2.5% of the total number of entries were aggregated into Others. Asterisks denote conformations not clustered according to PyIgClassify classification.

A total of 525 sdAb structures and 2,303 conventional mAb structures collected from SAbDab met our selection criteria (see *Methods*). We plotted the distribution of CDR configurations, *i.e.*, the respective lengths of the CDR-H1 and CDR-H2 loops that co-exist (**Figure 1**). Overall, more diversity in CDR configuration is observed for sdAbs than for IGHV3 and IGHV1 mAbs. The most prevalent CDR-H1–CDR-H2 configurations for sdAbs in the PDB are 13-10 and 13-9, accounting for 53% and 25%, respectively. These observations underline some previously reported data (4, 22). The subsequent analyses were entirely devoted to the dominant 13-10 configuration encompassing 276 entries for sdAbs, given the relatively low number of structures for other CDR configurations not nearly sufficient to infer statistically significant trends. While devoting efforts to 13-10 inevitably limited the scope of the study, it also channelled it towards human antibodies for which this configuration is most abundant and towards a more relevant space from a therapeutic perspective. The complete sets of conventional mAbs based on the IGHV3 and IGHV1 gene families encompass 669 (29%) and 819 (36%) structures, out of which 445 (67%) and 683 (83%) entries have the CDR-H1–CDR-H2 configuration 13-10. The final sets of “clean” structures contain 210, 315 and 524 entries for sdAbs, IGHV3 and IGHV1, respectively.

The distribution of antibody origins for sdAbs indicate a clear bias inherent to the Camelidae origin of sdAbs (**Figure 1**). Llama sdAbs account for 49% of the 13-10 entries followed by alpaca (20%) and camel (12%) while human dAbs account for 7% only. Eliminating this species bias is difficult due to limited availability of sdAb structures. However, throughout this study we make efforts in ensuring the observed trends do not emerge as a mere separation of species. A significant fraction of sdAb structures are sybodies (10%), *i.e.*, domain antibodies derived synthetically as a result of *in vitro* directed evolution (ribosome, phage or yeast-surface display). Other sdAbs were mainly obtained by immunizations of mice or nanomice. The IGHV3 mAb structures are mainly from human (88%), while IGHV1 mAb structures are from both human (44%) and mouse (47%).

The proportions in canonical clusters vary widely across the three classes: sdAbs, IGHV3 and IGHV1 (**Figure 1**). Unsurprisingly, the proportions are more similar between sdAbs and IGHV3 mAbs given their closer evolutionary proximity (14). The distribution of CDR-H2 canonical clusters for sdAbs mimic that of IGHV3 antibodies. Thus, sdAbs and IGHV3 mAbs display a relatively high preference for the canonical cluster H2-10-2 (57% *vs* 46%). In contrast, IGHV1 mAbs are dominated by the canonical cluster H2-10-1 (84%). Noteworthy, the closer similarity between sdAbs and IGHV3 mAbs does not apply to CDR-H1, where a smaller proportion of the canonical cluster H1-13-1 is observed for sdAbs than for conventional mAbs, corroborating previous data (4). As expected, the proportions in this study differ significantly from the averaged ones of North et al. that were calculated overall on merged antibody classes and, at the time of their publication, were 87% for H1-13-1, 68% for H2-10-1 and 19% for H2-10-2. The North et al. proportions are more similar to those for IGHV1 mAbs, the dominant antibody class in the PDB.

### Clustering Antibody Structures

A hierarchical clustering was performed separately for CDR-H1 and CDR-H2 from the set of antibody structures with CDR-H1–CDR-H2 configurations 13-10 and was limited to the better-resolved crystal structures. Ideally, each cluster would have its own sequence signature and could be uniquely described from a set of structural determinants. Towards that goal, a stringent threshold was applied to separate clusters with subtle differences in conformation to better capture the impact of sequence on structure, balance the size of clusters, and allow for small dynamic motions inherent at room temperature. The clusters can be represented as an ensemble of structures that fluctuate around a centroid structure. They are classified as major or minor clusters depending on the number of structures they contain (with a 5% threshold). To extract more general trends, the subsequent analyses focus more heavily on the major clusters. On average, minor clusters are less accurately defined and have larger fluctuations. The sdAb, IGHV3 mAb and IGHV1 mAb structure sets led to 6, 5 and 4 major clusters for CDR-H1, and 5, 5 and 5 major clusters for CDR-H2, respectively (**Table 1**; **Supplementary** **Tables 1, 2**). These major clusters covered 48% (sdAbs), 94% (IGHV3) and 75% (IGHV1) of the entries for CDR-H1, and 70% (sdAbs), 84% (IGHV3) and 80% (IGHV1) for CDR-H2; the rest of the structures were contained in minor clusters. The CDR-H1 sequence diversity was higher for the major clusters of sdAbs (~6 residues on average) than for conventional mAbs (~5 residues), while CDR-H2 sequence diversity was similar among the various antibody classes.

**Table 1:** Properties of CDR-H1 and CDR-H2 clusters for single-domain antibodies.

	Centroid structure	Topological variant^a^	Sequence diversity^b^		RMSF^c^		Count^d^				
			Loop	Paired	Average	Max	N	R71	A94	K_base_	IGHV
**CDR-H1**	5lmw[A]	H1-13-1.1.1	4.9 ± 1.8	6.2 ± 1.5	0.48	0.83	26 (12)	24 (92)	17 (65)	23 (88)	9 (35)
	3k74[B]	H1-13-1.1.2	4.6 ± 1.5	6.9 ± 1.5	0.32	0.73	18 (9)	18 (100)	0 (0)	11 (61)	15 (83)
	6ocd[D]	H1-13-1.2.1	6.6 ± 2.2	6.4 ± 1.9	0.60	1.56	15 (7)	15 (100)	12 (80)	12 (80)	0 (0)
	6z3x[B]	H1-13-1.2.2	6.3 ± 2.1	6.7 ± 1.9	0.46	0.91	12 (6)	7 (58)	1 (8)	6 (50)	4 (33)
	7kn7[B]	H1-13-1.2.3	6.3 ± 2.4	7.2 ± 1.7	0.59	1.65	12 (6)	11 (92)	8 (67)	12 (100)	0 (0)
	7n0i[K]	H1-13-5.1.1	6.5 ± 1.9	6.5 ± 1.4	0.43	0.89	16 (8)	9 (56)	14 (88)	14 (88)	0 (0)
**CDR-H2**	5m13[B]	H2-10-1.1.1	7.1 ± 1.5	7.9 ± 1.8	0.34	0.61	26 (12)	6 (23)	13 (50)	23 (88)	6 (23)
	5tp3[A]	H2-10-1.2.1	6.5 ± 1.2	7.1 ± 1.8	0.36	0.81	22 (10)	17 (77)	17 (77)	20 (91)	1 (5)
	5lhn[B]	H2-10-2.1.1	6.1 ± 1.5	5.6 ± 2.0	0.28	0.52	68 (32)	68 (100)	39 (57)	59 (87)	24 (35)
	6y0e[B]	H2-10-2.1.2	6.3 ± 1.5	6.9 ± 1.8	0.25	0.48	22 (10)	19 (86)	14 (64)	16 (73)	1 (5)
	1xfp[A]	H2-10-6.1.1	4.9 ± 1.3	6.2 ± 1.7	0.40	0.87	12 (6)	10 (83)	10 (83)	9 (75)	0 (0)

a The topological variant of the cluster as referenced by the predicted canonical structure according to PyIgClassify suffixed with the topology ID followed by variant ID. The canonical structure was obtained by finding the most frequent canonical structure among representatives of the cluster. The clusters were ordered by topologies and variants in terms of structure representation.

b Sequence diversity is quantified through a pairwise sequence comparison within representatives of a given cluster using a naive Levenshtein distance for the canonical loop of the respective cluster and the paired canonical loop (CDR-H2 for CDR-H1 clusters and CDR-H1 for CDR-H2 clusters).

c RMSF as calculated for B-factors of centroids.

d Count in number of representatives within the cluster (N), having Arg at position 71 (R71), having Ala at position 94 (A94), adopting a kinked CDR-H3 base geometry (K_base_) and deriving from a IGHV germline (IGHV). The percentages shown in parenthesis were calculated out of the total number antibody entries in the case of the N column, or out of the number of cluster representatives (N) in the case of the other columns.

The clusters were mapped to their respective canonical classes predicted by PyIgClassify. Several clusters correspond to the same PyIgClassify canonical structure, highlighting the higher stringency of our clustering criteria. For sdAbs, H1-13-1 is observed among all CDR-H1 major clusters except for one cluster that mapped to H1-13-5. The CDR-H2 major clusters map to the H2-10-1, H2-10-2 and H2-10-6 canonical structures (**Table 1**). For IGHV3, the major clusters of CDR-H1 fall into H1-13-1, while 5 major clusters of CDR-H2 were mapped to H2-10-2 and H2-10-1. Arg at position 71 (IMGT position 80) appears less predictive for the H2-10-2 canonical structure of sdAbs than of IGHV3 antibodies. In IGHV3 mAbs, its presence is strongly predictive of the H2-10-2 canonical structure, which is reflected in three IGHV3 CDR-H2 clusters, while the other two clusters lack Arg-71 and map to the H2-10-1 canonical structure (**Supplementary Table 1**). For IGHV1 antibodies, all major clusters fall into CDR-H1 and CDR-H2 canonical structures H1-13-1 and H2-10-1, respectively (**Supplementary Table 2**).

The IGHV and IGHVH germline origins were distinguished for the representatives of sdAb major clusters. The germlines were distinguished on the basis of the amino acid sequence at positions 44-46 (IMGT positions 49-51) that best corresponds to GLE (for IGHV origin) or ERE (for IGHVH origin). The counts and frequency of perceived IGHV origin are reported for every cluster (**Table 1**). An overall bias towards the IGHVH family is observed throughout the clusters with many of the clusters having exclusive representation in the IGHVH germline. Nonetheless, one CDR-H1 cluster shows a clear preference for IGHV.

Not to diminish the potential structural implications that CDR-H3 may have, clustering CDR-H3 with the objective of extracting similar structural determinants as done for CDR-H1 and CDR-H2 would not be possible provided its high sequence and structural variability. In truth, CDR-H3 has proven to be unique and unclassifiable (22, 31, 37). Instead, to assert the structural influence of CDR-H3 onto our clusters, the geometry of the base (or stem) residues was analyzed. The CDR-H3 was categorized as adopting either the kinked or extended geometries within the dihedral angle criteria previously proposed (41). Generally, a strong bias towards the kinked base geometry is observed, corroborating the results from a previous study (42). Most major clusters of sdAbs (**Table 1**), and of IGHV3 and IGHV1 mAbs (**Supplementary** **Tables 1, 2**) have frequencies of kinked CDR-H3 nearing 90%, with only two sdAb clusters having noticeably lower frequencies of 50% and 61%.

### Definition of Packing Pockets

**Figure 2:**
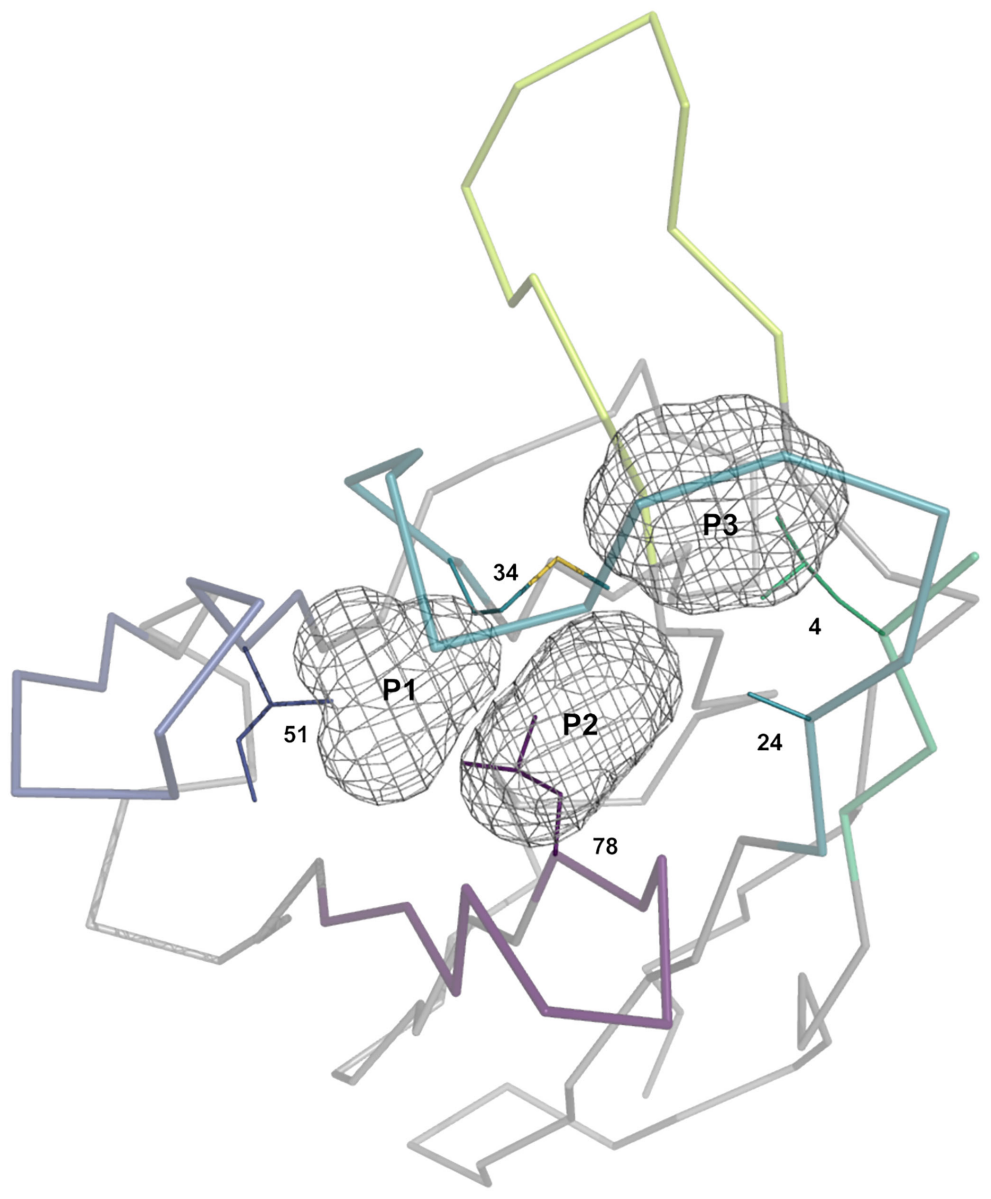
Representation of the three pockets (P1, P2 and P3) situated in the vicinity of the CDR-H1 (turquoise), CDR-H2 (blue), CDR-H3 (lime) loops, a segment of FR3 (purple) and a segment of the N-terminal (green). The pockets form, together with its surrounding residues, the core region that requires to be packed to avoid exposing core hydrophobic residues (numbered residues shown as thin solid lines). The core residues shown are the most probable at those positions: Ile-4, Ala-24, Met-34, Ile-51 and Ile-78.

To guide the visual description in the following sections, three pockets termed P1, P2 and P3 were defined (**Figure 2**). Due to the lack of CDR-H2, the P1 pocket is absent from V_NAR_ antibodies, thus making those unsuited for this analysis. The three pockets are in the vicinity of conserved hydrophobic residues composing a set of support residues deemed essential for the stability and structural integrity of the antibody structure. The support set includes residues Leu-4, Ala-24, Met-34, Ile-51 and Val-78 (IMGT positions 4, 25, 39, 56 and 87). Various regions of the antibody occupy these pockets: CDR-H1, CDR-H2, CDR-H3, a region of the framework (FR3) and the N-terminal region (NT). The pockets were defined from a reference structure (the representative 3k74[B]) that has the combination of the most frequently observed CDR-H1 and CDR-H2 canonical structures. The reference structure is viewed as an ideal and optimal state that fully satisfies the P1 and P2–P3 pockets due to the presence of conserved side-chains at key positions in the FR3 and CDR-H1.

Our study is based upon two premises. The first premise is that these pockets require to be packed to some extent to preserve structural integrity and that leaving the support set of residues fully exposed to the solvent would be detrimental energetically. In fact, the pockets are rarely seen left unoccupied (**Supplementary Figure 1**) with median percentages of occupancy for P1, P2 and P3 of 67%, 62% and 58%, respectively, for sdAbs structures. The second premise is that the various regions need to work together in order to fulfill the packing density of P1, P2 and P3 through a so-called *compensation* (as opposed to a *complementation*). Therefore, a change in a single residue in the framework or CDR sequence may result in the disruption of the reference state and in some form of *compensatory* structural changes leading to regions that are *complementary* to each other. Two sequences require to be *compatible* for two regions to be *complementary*.

### Description of Loop Topologies and Variants

Many of the clusters led to the same apparent canonical structure as defined by North et al. because of the stringent clustering criteria. Two scenarios could occur in such instances. (i) The clusters diverged sufficiently in sequence and structure, necessitating the division of the canonical structure into sub-populations, referred as topologies. In our classification, a topology is referenced by appending the topology ID to its inherited North et al. parent canonical structure, as in H1-13-1.1 for example. (ii) The clusters shared similar sequence and structural features, necessitating the division of the topology into variants. A topological variant in our classification is referenced by appending the variant ID to the topology, as in H1-13-1.1.1.

Differentiating loop topologies that co-exist within a single canonical structure helped us rationalizing specific sequence requirements important for driving the topology. The assignment of topologies was done qualitatively through visual inspection of the cluster representatives. Variants that are part of the same topology were ordered by cluster size with variant 1 having the largest number of cluster members. The major clusters are indicated in **Table 1** for sdAbs and **Supplementary Tables 1, 2** for conventional mAbs.

**Figure 3:**
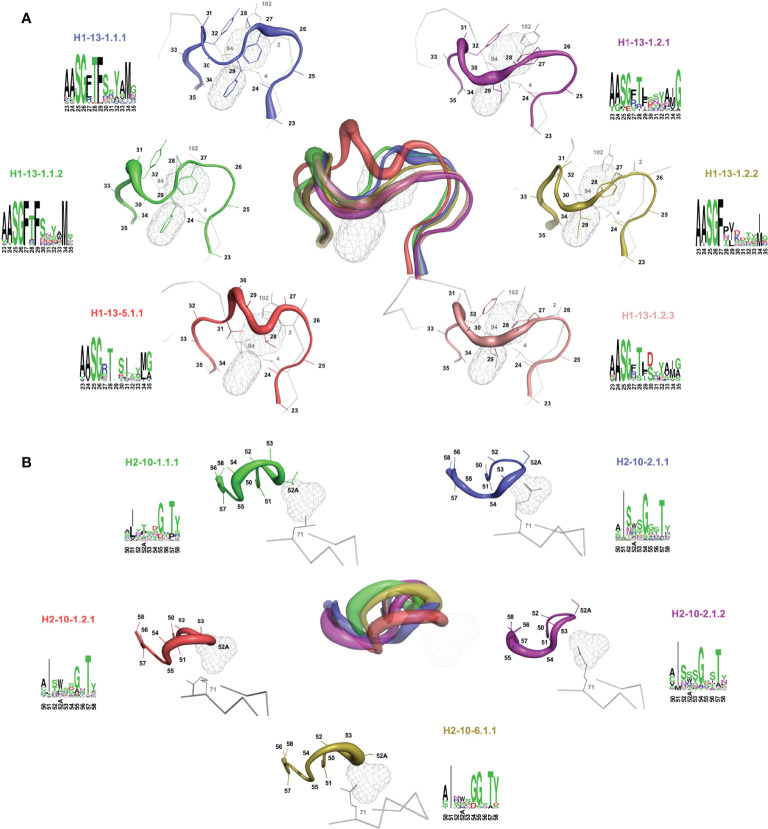
Rendering of the major topological variants for CDR-H1 **(A)** and CDR-H2 **(B)** for single-domain antibodies. CDR-H1 is displayed in the presence of pockets P2 and P3, and CDR-H2 in the presence of pocket P1 and residue at FR3 position 71. A superposition view depicts structural differences between the variants. The variants are uniquely colored and displayed individually as sausage views around their centroid structure (see **Table 1**). The centroid structures were superimposed based on the set of backbone framework atoms. The thickness of the sausage is indicative of the structural fluctuations among cluster members. Each variant is shown with its associated sequence logo with colors denoting the nature of the amino acids.

Sausage representations of the CDR-H1 and CDR-H2 major clusters were produced from the ensembles and were mapped onto their respective centroid structure as shown in **Figure 3** for sdAbs (see corresponding **Supplementary Figures 2, 3** for conventional mAbs). Only the relevant pockets are shown in the context of the CDR, *i.e.*, the pockets that are adjacent and frequently filled by the CDR loop in question. The sausage views accentuate segments of the CDR loops with more backbone atomic fluctuations upon structural alignment by representing them with more thickness. The major clusters have unique profiles in their burial of pockets P1, P2 and P3 for sdAbs (**Supplementary Figure 4**) and the IGHV3 and IGHV1 conventional mAbs (**Supplementary Figures 5, 6**), and show more similarities between variants within a topology than between topologies.

For a detailed structural interpretation of each major cluster of CDR-H1 and CDR-H2 loops for sdAbs as well as for the IGHV3 and IGHV1 conventional antibodies, the reader is directed to the **Supplementary Material**. In brief, for the dominant CDR-H1 canonical structure H1-13-1 we observe the two topologies H1-13-1.1 and H1-13-1.2 (**Figure 3A**). One distinguishing characteristic is the presence of Phe (H1-13-1.1) at position 29 (IMGT position 30) as opposed to a shorter aliphatic side-chain (H1-13-1.2) leading to a distinctive topology that brings CDR-H1 deeper towards P2. The H1-13-1.2 topology is exclusively observed in the sdAbs originating from the IGHVH germline and in the IGHV3 mAbs. The H1-13-1.1 (variants 1 and 2) and H1-13-1.2 (variants 1 and 2) topologies were observed in closed and open forms dictated by the nature of the residue at CDR-H3 position 94 (**Supplementary Figure 7**). The CDR-H2 canonical structure H2-10-1 was also divided into two distinct topologies H2-10-1.1 and H2-10-1.2, mainly driven by the nature of the residue at CDR-H2 position 52A (IMGT position 58) and framework position 71 (**Figure 3B**). A single topology was observed for the CDR-H2 canonical structure H2-10-2.

### Complementarity Between CDR-H1 and CDR-H2 Topological Variants

**Figure 4:**
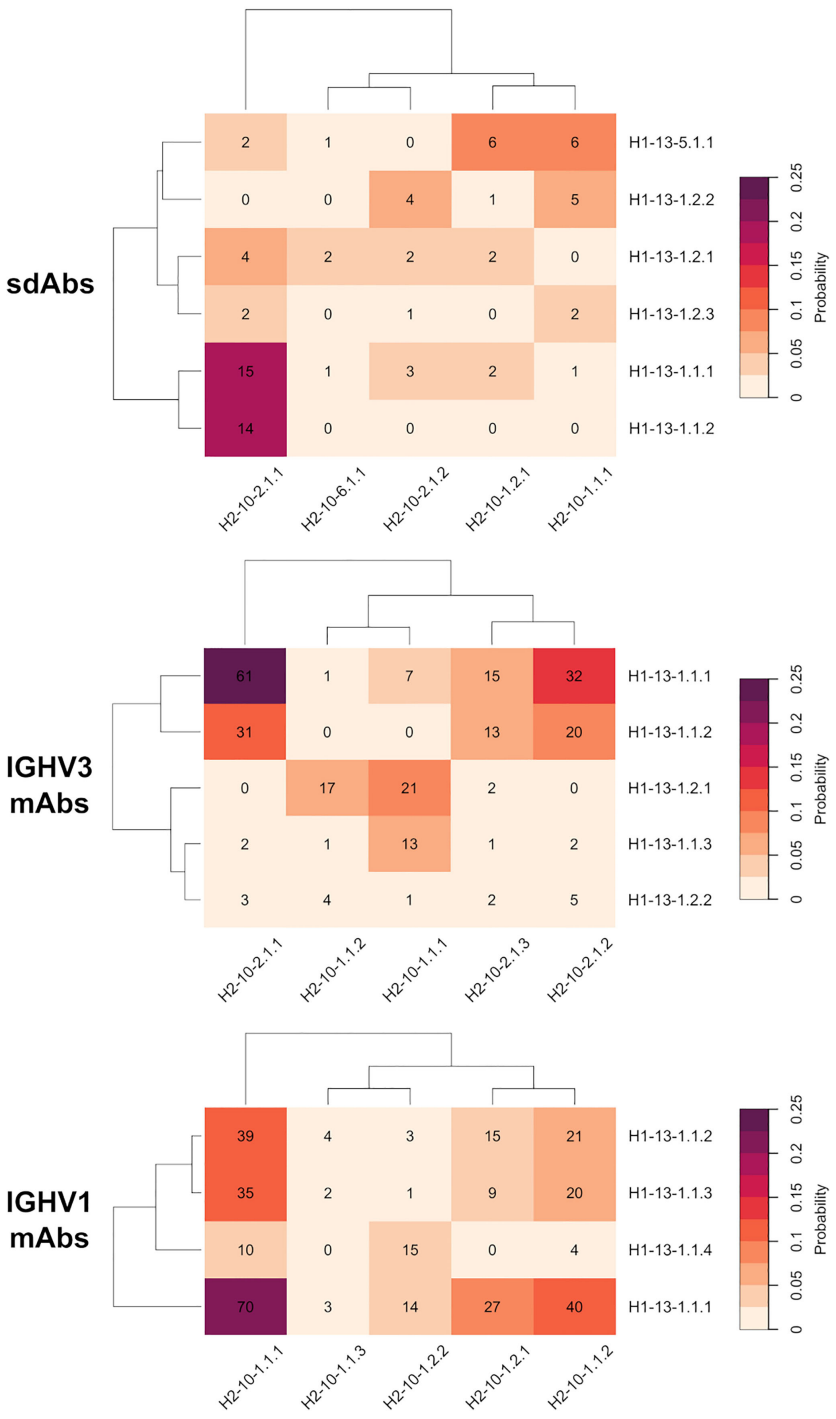
Probability heatmap of all combinations of major clusters of CDR-H1 against major clusters of CDR-H2 for the antibody classes under study: sdAbs, IGHV3 mAbs and IGHV1 mAbs. The probability scale displayed uses the following color scheme: from beige (not probable) to purple (probable). The probabilities are scaled to the total number of major cluster representatives. The number of representatives per combination is displayed in each cell.

Given the hypothesis of specific structural complementarity requirements between CDR-H1 and CDR-H2 sequences, we investigated potential biases in the combination of CDR-H1 and CDR-H2 topologies and variants. To this end, the frequencies of occurrence of every combination of CDR-H1 and CDR-H2 major clusters were calculated (**Figure 4**).

The CDR-H1 variants of the H1-13-1.1 topology having strong conservation of Phe-27 and Phe-29, are almost exclusively observed in presence of the CDR-H2 topology H2-10-2.1. The variant 1 (H1-13-1.1.1) and variant 2 (H1-13-1.1.2), representing the closed and open forms, have 100% and 82% probabilities of co-occurrence with H2-10-2.1, respectively. Interestingly, both the closed and open forms of this CDR-H1 topology co-exist more frequently with CDR-H2 topological variant H2-10-2.1.1 than with H2-10-2.1.2. This preference is striking in the case of the closed form H1-13.1.1.2 that exclusively prefers variant 1 over variant 2 of the same CDR-H2 topology H2-10-2.1. Of note, variants 1 and 2 differ by the nature of the residue at CDR-H3 position 94 (**Supplementary Material**) suggesting structural implications of the CDR-H3 in these conformational preferences. Such preferences for CDR-H1–CDR-H2 specific pairings could not be discerned with the broader canonical structures H1-13-1 and H2-10-2 available from PyIgClassify.

**Figure 5:**
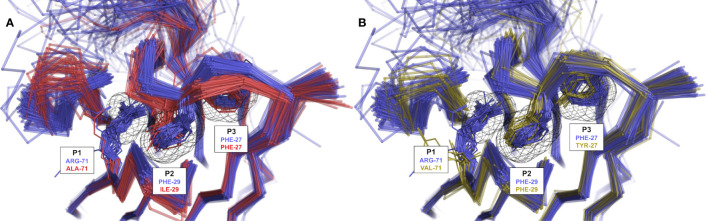
Rendering of population shift upon residue changes at structurally determining positions. **(A)** The presence of a short aliphatic side-chain (such as Ile) at CDR-H1 position 29 (H1-13-1.2.1 in red) leads to a topology change in which CDR-H1 moves deeper into P2 and exerts steric pressure onto P1 in contrast to Phe-29 (H1-13-1.1.1 in blue) observed in variants of IGHV3 mAbs. **(B)** The presence of Tyr at CDR-H1 position 27 (H1-13-1.1.3 in yellow) leads to similar steric pressure onto P1 in contrast to Phe-27 (H1-13-1.1.1 in blue) observed in variants of IGHV3 mAbs. The preference for non-Arg-71 frameworks is clearly visible in the presence of Ile-29 or Tyr-27. Some outliers were removed for visual clarity.

The three variants of CDR-H1 topology H1-13-1.2, introducing a shorter hydrophobic side-chain at position 29, are more diversely paired with CDR-H2 topologies H2-10-1.1, H2-10-1.2 and H2-10-2.1. Notably, the less heterogenous in sequence and better-defined variant 2 (H1-13-1.2.2) displays a lower propensity for Arg-71, thus highlighting a relationship between the presence of a short hydrophobic residue at position 29 and the absence of Arg-71 (**Table 1**). Lower propensities in Arg-71 can also be observed for topological variants H1-13-1.2.1 and H1-13-1.2.2 of IGHV3 antibodies (**Supplementary Table 1**). The selection of Ile at position 29 of these CDR-H1 variants impacts the set of compatible CDR-H2 and FR residues it can accommodate. The subtle rearrangement of the CDR-H1 topology *H1-13-1.2* deeper towards the P2 pocket exerts steric pressure onto the P1 pocket (**Figure 5A**). As a result, the overall structure is perturbed, with Arg-71 having to rearrange from its consensus packing state in the H1-13-1.1–H2-10-2.1 pairing described above. The presence of Tyr-27 also leads to a topology that exerts a similar steric pressure onto the P1 pocket (compared to when Ile-29 is present) and contributes to the selection of the non-Arg-71 frameworks (**Figure 5B**). Although these changes allow more diverse pairing opportunities for the CDR-H1 topology H1-13-1.2 than H1-13-1.1, distinct preferences are still observed. For example, the H1-13-1.2.2 variant has a pairing preference for H2-10-1.1.1 and H2-10-2.1.2 but excludes pairings with H2-10-2.1.1 and H2-10-6.1.1. The pairing preference for H2-10-2.1.2 over H2-10-2.1.1 can be rationalized from the steric pressure of Ile-29 onto P1 forcing CDR-H2 to adopt a topology that can accommodate rotations of Arg-71 (complete description of variants given in the **Supplementary Material**).

The CDR-H1 cluster within the H1-13-5.1 topology is mainly observed in the presence of CDR-H2 topological variants H2-10-1.1.1 and H2-10-1.2.1. In the H1-13-5–H2-10-1 combination, the P2 pocket can no longer be filled by CDR-H1. Hence, CDR-H2 sequences evolved to compensate for the large unoccupied volume within P2. In some cluster members, CDR-H2 evolved towards bulky side-chains at position 52A to fill the P1 pocket (for example Trp-52A). In other instances that have shorter side-chains at position 52A (Ile, Pro or Thr), unconventional framework sequences or the presence of well-ordered water molecules were required to fill the void in the P2 pocket. In extreme cases, which are not the result of natural selection but represent synthetic antibodies, the P2 pocket cavity remained empty.

We observed preferences in heavy-chain CDR-H1–CDR-H2 pairings also for the IGHV3 and IGHV1 classes of conventional antibodies, suggesting generality of evolutionary selection towards dominant CDR conformations (**Figure 4**). While there are certain similarities with the pairing of topological variants of sdAbs, independent analyses of the various antibody classes were required for a clear understanding of pairing preferences in each class. This was further imposed given that analysis of conventional antibodies may be confounded by other structural constraints imposed by their light-chain CDR loops. For example, the three variants within topology H1-13-1.1 for IGHV3 mAbs show preferences in their pairings. Variant 2 (H1-13-1.1.2) is exclusively observed in presence of H2-10-2.1 while variant 1 (H1-13-1.1.1) can additionally support H2-10-1.1. On the contrary, variant 3 (H1-13-1.1.3) has a strong preference for H2-10-1.1 and has the lowest propensity for Arg-71 out of all three variants (**Supplementary Table 1**).

### Game of Musical Chairs Between CDR-H1 and CDR-H2 Topological Variants

We quantified the volume occupancies of the P1, P2 and P3 pockets for all observed combinations of CDR-H1 against CDR-H2 clusters. In absolute terms, 50% of the CDR-H1–CDR-H2 combinations fall within the 47%-63% range of combined occupancy of all pockets, P1+P2+P3 (90% of CDR-H1–CDR-H2 combinations have 30%-80% combined occupancies). The median combined occupancy was 58%, indicating that full occupancy of the pockets is not a requirement to preserve good stability (**Supplementary Figure 8**). We compared those occupancies with those of the reference structure having the most frequently observed canonical pairing H1-13-1.1–H2-10-1.1 and also having full occupancy of three pockets. We investigated if a loss in occupancy relative to the reference structure concurrently results in a structural compensation (and at what extent a loss must be compensated). This would underline a game of musical chairs that needs to happen to preserve structural stability.

**Figure 6:**
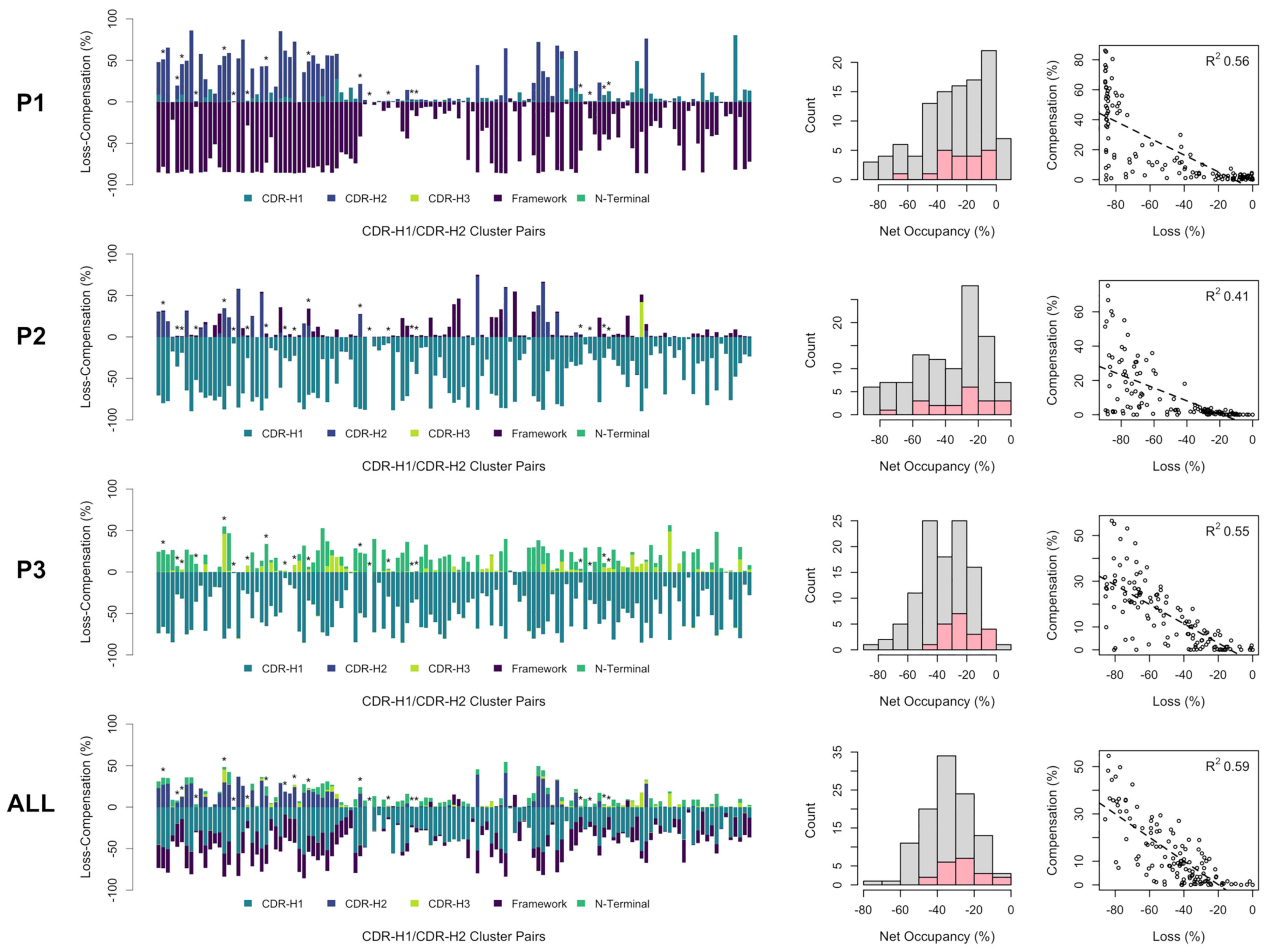
Occupancies for all observed CDR-H1 and CDR-H2 cluster pairs of single-domain antibodies (in no particular order). The loss (negative) and compensation (positive) in occupancies are plotted (bar plots on the left) for the various regions that can occupy the pockets P1, P2, P3 and all three pockets combined. A template structure that fully occupies the pockets is used as reference (PDB: 3k74[B]). Major clusters are labelled with asterisks. Net occupancies (loss + compensation) for all cluster pairs are counted (middle histograms), with the major cluster pairs shown in pink. The correlations between the loss and compensation in occupancies for all cluster pairs are shown (scatter plots on the right) with associated Pearson coefficients (R^2^).

The incurred losses and accompanying compensations in the occupancies of individual as well as combined pockets P1, P2 and P3 are shown in **Figure 6** for all combinations of the sdAb clusters. As a general observation, the losses in occupancy are indeed compensated but at lower levels relative to the reference structure. Nevertheless, we observed good correlations of loss-*versus*-compensation, with squared Pearson coefficients (R^2^) of 0.56, 0.41 and 0.55 at P1, P2 and P3, respectively. The compensation game of musical chairs is more noticeable for cases experiencing larger losses. The correlation plots appear to suggest that some amount of P1, P2 and P3 volume can be lost without requiring any compensation up to a critical level (~20% loss). There are several cases where near-complete volume loss in either P1, P2 or P3 does not result in significant compensation in these individual pockets (see outliers on the bottom-left regions of the plots). This highlights that only limited structural information can be extracted by looking at individual pockets in isolation and that one should view the packing problem more globally. In fact, a higher correlation with an R^2^ coefficient 0.59 and fewer outliers is achieved when combining data for all three pockets. The correlation plot over combined pockets (ALL in **Figure 6**) tends to suggest more clearly that 20% of combined occupancy can be lost without requiring compensation. Despite their higher number of structures that is putatively indicative of increased thermodynamic relevance, the distribution of net occupancies for pairings of major clusters closely mimics that for pairings between minor clusters (see histograms in the middle column of **Figure 6**).

## Discussion

### Narrow Clusters can Provide Novel Insights Into Sequence-Structure Relationships

One objective of the current study was to capture some of the subtle structural differences that may occur from residue changes in one canonical CDR loop sequence and to relate that to changes in the structure of other parts of the CDR and the framework. To capture the changes, a naive clustering approach based on RMSD was employed. A meticulous visual inspection of the clusters was carried out to obtain a better understanding of the sequence-structure relationships. By doing so, we hoped to identify key sequence-structure determinants that drive CDR loops into specific topologies. In the ideal scenario, each cluster would have distinct sequence-space coverage for the positions deemed important for the structural integrity of these topologies. Many challenges can arise during clustering, with some being inherent to clustering algorithms. For example, some data points falling on the edges of the clusters can be more difficult to assign to one cluster or another and can blur trends. In fact, some clusters had no clear separation in sequence-space and thus had mixed topologies, *e.g.*, the variants H1-13-1.2.1 and H1-13-1.2.3 for sdAbs. This could arise from multiple factors such as small conformational changes at room temperature or sequence variations of the CDR loop in question. Also, our clustering methodology did not factor in residue changes in the CDR-H3 or framework. Therefore, some structural changes observed between clusters might not necessarily result from a residue change in the clustered CDR loop in question but could result from compensation by a structural change elsewhere. However, this had the benefit of identifying multiple clusters covering the same sequence-space in cases where the CDR loop conformation underwent compensatory structural changes (*e.g.*, open and closed forms).

The choice of the clustering threshold value was another challenge. It impacts the number and size of the clusters and hence it directly affects the sequence-space coverage of each cluster. Here, the threshold was set empirically with criteria that met our objectives while providing a balance between sequence-space coverage and data representation. While there are benefits in using permissive thresholds for data representation, doing so would not allow detection of subtle but important conformational changes and it would replicate the canonical clusters from North et al. having a relatively wide sequence-space coverage. Instead, we leaned towards a more stringent threshold in an attempt of identifying clusters within narrow regions of the sequence space, which were then back-linked to the wider canonical structures previously proposed. Stringent clustering allows us to capture subtler conformational preferences, which we hypothesized to be sufficient to trigger a series of propagated structural changes into the rest of the structure that would have meaningful implications. However, there were downsides of using a stringent threshold. Some of the clusters that were defined cover similar sequence spaces and were separated simply due to normal structural fluctuations at room temperature. For instance, many minor clusters of sdAbs have conserved Arg-27 and Phe-29 but were separated on the basis of CDR-H1 residues 23-27 alone, which had increased fluctuations in various crystal structures (as indicated by higher B-factors).

Efforts were made to ensure that observed sequence-structure relationship trends were general enough and were not a result of sporadic events (*e.g.*, unconventional framework sequence, rare CDR sequence). Thus, we focused primarily on clusters that have at least 5% representation. CDR sequence diversities were quantified to ensure results do not emerge from redundant sets of sequences. This rigorous approach led to robust trends characterized by strong underlying signatures driven by a few residues at key positions. The clusters vary on average at 6 out of 13 residue positions in CDR-H1 (45% variance) and 6 out of 10 positions in CDR-H2 (60% variance). A compromise on data representation was made to remove low-populated clusters. Therefore, the scope of our conclusions is limited to those sequences with better representation in the PDB. By devoting most efforts to major clusters of CDR-H1 and CDR-H2, several architectures of canonical structures may have been missed. Of note is the case of sdAbs, which is peculiar since they have the highest CDR sequence diversity but the smallest number of entries in the PDB among the antibody classes compared here.

### Canonical CDR-H1–CDR-H2 Sequences Evolved Towards Complementarity to Framework

It was previously observed that the set of CDR-H2 canonical structures is heavily influenced by the nature of the framework residue at position 71. A reasonable approach is that CDR-H2 canonical class could be predicted on the basis of the FR3 position 71 alone. Indeed, only CDR-H2 and/or position 71 can occupy a significant fraction of an adjacent pocket (called P1). However, that approach is based on a very localized feature, and one should look at the bigger picture that includes the whole antibody domain structure. In this study, we noticed some CDR-H1 canonical structures that appeared to have a remote structural relationship to Arg-71. Hence, we extended the position 71 based approach to include other structural features and defined two other connected pockets (called P2 and P3). The pockets were employed to determine which regions occupy the empty space needed to be packed in the vicinity of support residues in order to preserve structural integrity. With P2 connecting CDR-H1 and CDR-H2, and P3 connecting CDR-H1 to CDR-H3, the three pockets served as proxy in detecting some of the compensatory structural changes that are required to account for sequence changes. For instance, we observed a structural compensation in cases where CDR-H1 applies steric pressure onto P1 due to sequence changes at CDR-H1 positions 27 and 29. This led to disposal of the Arg-71 framework side-chain either by mutation or by conformational change away from P1 and into the solvent. The evolutionary selection of the residue at position 71 appears thus not as a localized event but it rather encompasses a more widespread complementarity requirement across the whole domain structure. Our results here reveal such structural complementation, which are transcribed into specific repertoires of permitted combinations of CDR-H1–CDR-H2 topologies.

The combinations observed likely emerged during evolution to preserve folding stability while allowing for CDR diversity. In this respect, the H1-13-1.1–H2-10-2.1 combination may be regarded as having the highest thermodynamic stability for sdAbs and related IGHV3 antibodies given its highest frequency. This combination achieves an exceptional packing of all three pockets, P1 by Arg-71, P2 by Phe-29 and P3 by Phe-27. Mutation of framework Arg-71 would leave the P1 cavity open and lead to H1-13-1.1–H2-10-2.1 structure destabilization. This may have required natural selection of another canonical structure, H2-10-1, to re-establish the strong packing within P1. There was probably a strong evolutionary selective pressure to retain Arg-71 and the compatible H2-10-2 canonical structure for optimal packing within P1. This is especially the case of sdAbs, where the only two known structures with H1-13-1.1 topology that do not feature Arg-71 have acquired another CDR-H2 topology, H2-10-1.1, instead of the typical H2-10-2.1. On the other hand, IGHV1 structural evolution took a different path and focused instead on the CDR-H1 position 27 where Tyr was selected. Selecting for Tyr-27 disturbs packing, minimizes the requirement for Arg-71 and acquired a preference for the matching CDR-H2 topology H2-10-1.1. Obviously, not all theoretical combinations of CDR-H1–CDR-H2 canonical sequences and topologies are energetically equally stable. In extreme cases, some CDR topologies, if combined, would likely lead to steric conflicts. For instance, positions 32 and 52A would clash in the CDR-H1–CDR-H2 pair H1-13-5.1–H2-10-2.1. To relieve the steric conflict, CDR-H1 topology H1-13-5.1 evolved towards complementarity with the CDR-H2 topology H2-10-1.1, another pair that affords a stable fold by avoiding leaving pockets P1 and P2 empty. The natural evolution of specific pairings between CDR-H1 and CDR-H2 canonical topologies can thus be mirrored by the game of musical chairs, where CDR-H1, CDR-H2 and FR complement each other to solve the packing problem.

### sdAb CDR-H1–CDR-H2 Paratopes Are More Variable Than Those of Conventional IGHV3 or IGHV1 mAbs

Previous studies tend to have suggested that sdAbs display higher structural variability in their paratope in comparison to conventional mAbs (4, 7, 43). From an evolutionary perspective, it would make intuitive sense that sdAbs would require higher diversity, to offset for the lack of the light chain present in conventional mAbs. It has been shown that one mechanism that sdAbs employ to gain diversity is from having longer hypervariable CDR-H3 loops. Our study illustrates some other mechanisms employed to gain diversity, most particularly arising from the CDR-H1 and CDR-H2 canonical loops. The results support the notion that conventional mAbs display a limited diversity in canonical structures and most particularly in their CDR-H1 loop (**Table 1**; **Supplementary Tables 1, 2**; **Figure 3**; **Supplementary Figures 2, 3**). On the other hand, CDR-H2 diversity stays more-or-less conserved from sdAbs to related IGHV3 mAbs, while IGHV1 mAbs show less diversity.

Not only do sdAbs have an overall higher sequence diversity, that diversity is more prominent at some of the positions that were shown to be structurally determining, which indirectly gives rise to more combinations between canonical CDR-H1–CDR-H2 topologies. In human antibodies from the IGHV3 family, the residues Phe-27, Phe-29 and Arg-71, along with framework residues, are all more conserved and do not allow for as much structural variability. Similarly, IGHV1 mAbs display a strong conservation of Tyr-27 and Phe-29, which constrain the set of CDR-H1–CDR-H2 canonical combinations that can exist. In contrast, more sequence diversity is observed for sdAbs at those two crucial positions, including: (i) increased propensity for shorter hydrophobic side-chains at position 29 leading to a CDR topology that can tolerate both Arg-71 and non-Arg-71 frameworks; (ii) higher propensity for Arg-27 leading to CDR-H1 flexibility given the high exposure of this side-chain; (iii) more sequence variability at position 94 that can trigger open and closed forms of the CDR-H1; (iv) increased variabilities at some of the support residues thus expanding the canonical repertoire; and (v) novel canonical structures not present in conventional mAbs, such as H1-13-5. While part of the sequence diversity could be explained by the germline origin, our study provides a complementary view for the relative plasticity of the sdAb paratope apart from the hypervariable CDR-H3 loop.

### Modeling Templates for sdAb CDR Canonical Loops

One thing this study demonstrated is that subtle movements may have larger structural implications than initially thought and that such subtleties should be factored in for antibody design. One may be tempted to use the centroid of the canonical clusters to model canonical CDR loops. For instance, modeling a representative of H1-13-1.2 using as reference structure the centroid of the larger H1-13-1 canonical class from PyIgClassify (which in fact reflects the H1-13-1.1 topology) may not be the best decision. Instead, one should opt for a H1-13-1.2 template to ensure a proper packing of the CDR with the support residues. Not doing so would lead to inaccuracies in the predicted canonical structures and propagate errors in the remainder of the structure and ultimately hinder model quality. Our study underlines the necessity of better-suited structural templates for canonical sequences to produce higher-quality models. The centroid structures of the sdAb CDR-H1 and CDR-H2 canonical clusters generated in this work are listed in **Table 1**.

## Data Availability Statement

The original contributions presented in the study are included in the article/**Supplementary Material**. Further inquiries can be directed to the corresponding author.

## Author Contributions

FG and TS conceived the research plan and FG carried out the bulk of the computational work. All authors contributed to writing and reviewing the manuscript.

## Conflict of Interest

The authors declare that the research was conducted in the absence of any commercial or financial relationships that could be construed as a potential conflict of interest.

## Publisher’s Note

All claims expressed in this article are solely those of the authors and do not necessarily represent those of their affiliated organizations, or those of the publisher, the editors and the reviewers. Any product that may be evaluated in this article, or claim that may be made by its manufacturer, is not guaranteed or endorsed by the publisher.
