# Supplementary material for: Coevolved Canonical Loops Conformations of Single-Domain Antibodies: A Tale of Three Pockets Playing Musical Chairs
Source: Front Immunol. 2022 Jun 3;13:884132. doi: 10.3389/fimmu.2022.884132 (PMC9203998; doi:10.3389/fimmu.2022.884132)
Supplement: Supplementary file 1 [file DataSheet_1.pdf]

Supplementary Material

1 Supplementary Methods

NCBI Protein BLAST was used in finding a non-redundant set of antibody sequences that matched specific template sequences using the default algorithmic parameters (1). The BLAST hits were further filtered to only include sequences with CDR configuration 13-10. A single entry was retained for every unique heavy-chain canonical CDR sequence (CDR-H1–CDR-H2). BLAST searches were performed separately for the taxonomic IDs 9606 (*Homo sapiens*), 9844 (*Lama glama*) and 30538 (*Vicugna pacos*). The sequences of PDB structures 1oaq[H] and 5lmw[A] were used as template sequence in the BLAST search for IGHV1 for taxonomic ID 9606 and IGHV3 for taxonomic ID 9606, 9844 and 30538.

2 Supplementary Text

2.1 Description of loop topologies and variants

2.1.1 sdAb CDR-H1 topological variants

The CDR-H1 loop topology H1-13-1.1 was the most frequent, accounting for 21% (44 sdAb structures). The variants of this topology have the lowest sequence variability among all major clusters. The topology H1-13-1.1 is characterized by two hydrophobic side-chains at positions 27 and 29 having their Cβ atoms pointing towards CDR-H3 and FR3. The topology displays strong conservation of Phe at both positions (**Figure 3**) that form a relatively sharp angle and fill pockets P3 and P2. The good packing provided by Phe does not require compensation from the neighbouring regions. In a few cases, Leu replaces Phe-27. On average, Phe-29 fills well the P2 pocket of variant 1 (H1-13-1.1.1 with 77%) and variant 2 (H1-13-1.1.2 with 88%) without requiring compensation from the neighbouring regions, and Phe-27 fills the P3 pocket of variant 1 (58%) and variant 2 (82%) with minimal compensation from CDR-H3 (**Supplementary Figure 4**).

One characteristic of variant 1 (H1-13-1.1.1) is the high propensity of Ala at CDR-H3 position 94 as opposed to variant 2 (H1-13-1.1.2) that more predominantly requires positively charged residues Lys and Arg at this position. Variant 2 is notably more frequent in the IGHV germline origin suggesting a close relation with Lys/Arg-94. Interestingly, the predominance for Lys/Arg-94 is associated to a decreased in CDR-H3 kinked conformation. The impact on structure is noticeable when comparing the two variants (**Supplementary Figure 7**). The presence of the short side-chain of Ala is accompanied by a collapse of the surrounding side-chains at position 32 and 102 (mainly Tyr) onto the P3 pocket leading to an apparent closed form of the H1-13-1.1. Inversely, the topology shows more fluctuations at position 32 to adopt a more open form in the presence of the longer side-chains of Lys/Arg-94. An important structural rearrangement that moves CDR-H3 away from CDR-H1 to readjust the Cβ at position 94 is required to avoid clashing with Phe-27. The closed and open forms explain some of the differences between variants 1 and 2 in terms of pocket P3 packing. Side-chains at position 28, 30, 31 and 32 are exposed to solvent. Tyr is the most frequently observed residue at position 32 and is inserted between CDR-H1 and CDR-H3. The closed form prefers Tyr-32 which may explain its collapse for maintaining structural integrity, while the open form is more tolerant at position 32. The position 30 falls on the periphery of the paratope and prefers Ser. This is consistent with previous observations that the paratope periphery is predominantly populated with short hydrophilic residues deemed necessary for antibody specificity (2). Thr is

almost exclusively observed at position 28. The most variable position 31 exposes mainly polar side-chains.

The second most prominent topology, H1-13-1.2, is one that closely matches the one of the H1-13-1.1 topology and that has 3 variants (**Figure 3A**). The topology is almost exclusively observed in the IGHVH germline for the 3 variants. For variant 2 (H1-13-1.2.2), most members are in the open form, which also features two hydrophobic side-chains at position 27 and 29 but is characterized by a predominance for shorter hydrophobic residues (mainly Leu, Ile and Val) at position 29. This requires the Cβ at position 29 to face towards P2, instead of facing towards FR3 which was seen for Phe-29. The presence of the shorter hydrophobic residue leads to structural changes involving the CDR-H1 loop to move deeper towards the support residues of the pocket P2 (**Figure 5A**). The packing of P2 is still fulfilled mainly by position 29 but to a lesser extent (61%) than in H1-13-1.1. These structural changes are accompanied by a topology change that moves CDR-H1 further away from CDR-H3 leading to a widening of the angle between residues 27 and 29. The change does not influence the packing of P3 by Phe-27 (73%).

The variants 1 (H1-13-1.2.1) and 3 (H1-13-1.2.3) are more difficult to interpret due to higher sequence variability and more heterogenous topologies (**Figure 3A**). A characteristic that differentiates them from variant 2 is the location of the shorter hydrophobic side-chain at position 29. In variant 2, this side-chain approaches closer the P1 pocket than in variants 1 and 3. Some other representative structures within variants 1 and 3 having Phe-29 display an Arg at position 27. Because Arg-27 does not contribute to P3 packing, Phe-29 often fulfills this role by rotating towards P3. The presence of Arg-27 certainly adds to the difficulty of successfully modeling CDR-H1 given the uncertainties in correctly modeling a residue that can move freely into the solvent. In fact, the clustering criteria led to many distinct minor variants that have a preference for Arg-27, with significant changes in Cβ orientation at position 27 required for accommodating Arg-27. In some cases, these changes required compensation from the N-terminal residue Val-2.

Lastly, one cluster adopts an entirely different topology than the H1-13-1 topologies and that is exclusively observed in the IGHVH germline. It adopts the H1-13-5.1 topology that exposes the side-chains at position 27, 29 and 30 and buries positions 28 and 31. The heavily conserved residues at positions 27 and 29 in H1-13-1 are no longer conserved in H1-13-5.1. Instead, positions 28 and 31 are more heavily conserved. Position 28 displays a high propensity for β-branched side-chains Thr and Ile. Another distinction of H1-13-5 from H1-13-1 is the flipped orientation of the residue at position 32. Importantly, the H1-13-5.1 topology shows a unique packing signature in the P2 and P3 pockets with larger portions of P2 and P3 being left unoccupied (**Supplementary Figure 4**). The CDR-H1 residue at position 27 can no longer fill the P3 pocket. Instead, that role must be assumed by residue 31 and neighbouring regions. The P3 is only filled by the CDR-H1 (20%) and the N-terminal region (29%). The presence of Tyr-102 appears to be structurally important to prevent the exposure of the P3 pocket and shield it, together with Leu-4 from the N-terminal region. This compensation is facilitated by a strong propensity for Ala-94. In addition, CDR-H1 residue at position 29 can also no longer fill P2. Instead, we observe that CDR-H2 helps to partially fills the P2 pocket. These observations indicate that CDR-H1, CDR-H2 and other regions need to work together and suggest some compatibility in their sequences to solve the overall packing problem.

2.1.2 sdAb CDR-H2 topological variants

Two CDR-H2 clusters adopt the canonical structure H2-10-2, characterized by an 'S' shape from a bird's eye view where the CDR sits on top (**Figure 3B**). Combined over both clusters, it is the most frequent topology, accounting for 42% (90 sdAb structures) (**Table 1**). Distinctively in this topology, the residue at position 52A has its Cβ atom pointing onto the paratope surface ready for antigen interaction. The strongly conserved Arg-71 from FR3 is buried underneath CDR-H2 into a conserved

position to best fill the P1 pocket. The neighbouring residues at position 52, 53, 55 and 56 all expose their side-chains onto the surface. Many of those are pointing towards each other which may potentially cause obstruction at the surface. This may explain the preference for short hydrophilic side-chains at these positions (**Figure 3B**). A short side-chain, preferably Gly, is required at position 54 to avoid steric conflicts with the residue Arg-71. We describe this topology as H2-10-2.1. In variant 2 (H2-10-2.1.2), the angle formed by the Cα of residues 50, 51 and 52 is sharper, allowing CDR-H2 to move further away from P1 and hence open up this pocket. This change in conformation does not appear to be driven by a specific residue as the sequence conservation for the two variants are nearly the same with both variants having full conservation of Arg-71 as well. Notably, the change in conformation is necessary to allow Arg-71 to rotate freely out of its consensus position but still occupy P1. FR3 contributes less to P1 in variant 2 (48%) than variant 1 (77%). In addition, compensatory structural changes from CDR-H1 are required (**Supplementary Figure 4**).

Three CDR-H2 clusters adopt the canonical structure H2-10-1, characterized by a 'U' shape topology (**Figure 3B**). As opposed to H2-10-2, the side-chain at position 52A is buried with its Cβ oriented towards P1. The conserved Arg-71 is most often seen flipped out to accommodate the residue at 52A. In fact, the P1 pocket is mainly occupied by CDR-H2 (**Supplementary Figure 4**) for all 3 variants (44%, 36% and 34%). The residue at position 55 is outside of the paratope and no longer accessible for antigen interactions when compared to H2-10-2. Short side-chains are preferred at that position with Gly being preferred most, as long side-chains would conflict with the flipped-out state of Arg-71. The angle formed by the Cα of residues 52A, 53 and 54 is sharper for topology H2-10-1.1 than for topology H2-10-1.2 that displays a wider 'U' shaped. Some of the sequence characteristics that distinguish H2-10-1.2 from H2-10-1.1 are the increased propensities in Arg-71 and Trp-52A. Notably, the H2-10-1.1 topology has the lowest fraction of Arg-71 (23%) while the H2-10-1.2 topology has 86%. There is a higher occupancy of P1 by FR3 for H2-10-1.2 with 15% and 18% than for H2-10-1.1 with 6%. Despite having a structurally similar topology, one variant adopts the unique canonical structure H2-10-6 with flipped orientation of backbone atoms at position 53.

2.1.3 IGHV3 topological variants

All major CDR-H1 clusters for IGHV3 mAbs are mapped to the PyIgClassify H1-13-1 canonical structure, with all variants exclusively observed in the open form given the high frequency for Lys/Arg at position 94 (**Supplementary Table 1**). The trends observed for IGHV3 confirm the ones observed for sdAbs but with stronger statistical significance. Three variants have the H1-13-1.1 topology with strong conservation of Phe at positions 27 and 29 (**Supplementary Figure 2A**). The structural differences in CDR-H1 between variant 1 and 2 can be attributed in part to the stronger conservation of Tyr at CDR-H3 position 102 for variant 1. The presence of Val-102 in variant 2 forces the N-terminal region to move further outside leading to movements of CDR-H1 and CDR-H3 side-chains in the same direction. The presence of Tyr-27 in variant 3 also leads to the same H1-13-1.1 topology. However, the hydroxyl group of Tyr needs to be accommodated by the side-chain of Phe-29 that moves further away from P2 and more closely to P1 (**Figure 5B**). This is reflected by the lower P2 occupancy in by variant 3 (75%) than for variants 1 and 2 (94 and 95%) (**Supplementary Figure 5**).

Two clusters are mapped to the H1-13-1.2 topology having strong conservation of short hydrophobic side-chains at position 29. Both clusters have increased preferences for Ile at position 34 (**Supplementary Figure 2A**). The variant 2 (H1-13-1.2.2) has members with Phe-29 that still follow the H1-12-1.2 topology. This exception to the rules previously described can be explained, in most instances, by the presence of a framework sequence with unconventional support residues. That is, Phe-29 compensates a cavity created by shorter hydrophobic side-chains (such as Val, Thr or Ala) at position 34 replacing the usual longer hydrophobic side-chain (such as Met or Ile).

Three CDR-H2 clusters are mapped to the 'S' topology H2-10-2.1 (**Supplementary Table 1**). In nearly all instances of H2-10-2.1, Arg-71 occupies its consensus position into P1 (occupancies of 80%, 80% and 72% for the three respective variants) (**Supplementary Figure 5**). Most structural variations between these variants are along the stretch of residues 54 to 57 (**Supplementary Figure 2B**). Some of the variations can be explained by a higher propensity for Ile at position 57 in variant 2 (H2-10-2.1.2) than Thr in the other variants, which pushes CDR-H2 farther away from FR3 without affecting the residues upstream of position 54. Position 54 can better tolerate Ser at position 54 instead of the usual Gly in the presence of larger Ile-57. The variants 1 (H2-10-2.1.1) and 3 (H2-10-2.1.3) follow more closely the topology of sdAbs given their higher frequency for Thr-57 which does not interfere as much with CDR-H2. The structural differences between variants 1 and 3 could not be attributed to one specific residue and appear to be the result of intrinsic motions of the CDR-H2 arising from the nature of the residues at positions 53 and 55 facing each other onto the surface.

The remaining two variants are mapped to the 'U' topology H2-10-1.1 (**Supplementary Table 1**). Relative to variant 2 (H2-10-1.1.2), variant 1 (H2-10-1.1.1) is further rotated inwards to point the Cβ of residue 52A towards P1 and better fill this pocket. In fact, the P1 pocket is filled by CDR-H2 at 59% and 21% as well as by CDR-H1 at 3% and 10% occupancies for variants 1 and 2, respectively (**Supplementary Figure 5**). These variants do not have propensity for Arg-71 that is no longer needed to contribute to P1. One characteristic of IGHV3 mAbs is the high predominance for Pro at position 52A, which is more rarely seen in sdAbs. Pro-52A is most frequently observed in variant 1, where it fills a good portion of the P1 pocket (59%).

2.1.4 IGHV1 topological variants

The four variants of CDR-H1 in IGHV1 mAbs are mapped to the topology H1-13-1.1 and all have similar sequence variabilities (**Supplementary Table 2**). The variant 1 (H1-13-1.1.1) is better defined and displays less fluctuations than the other three variants. Like variant H1-13-1.1.3 of IGHV3 mAbs, Tyr almost exclusively occupies position 27 for all variants of IGHV1 except for variant 4 (H1-13-1.1.4) that also has representatives with Phe-27 at lower frequency (**Supplementary Figure 3A**). Position 33 is frequently occupied by Trp for variants 2 (H1-13-1.1.2) and 3 (H1-13-1.1.3). Position 30 is less variable than in other antibody classes and is often occupied by Thr. The structural differences between the variants appear to be the result of intrinsic motions of the CDR-H1 loop but could not be attributed to specific residues in sequence. Some of the differences may occur from subtle variations at positions 33 and 35. For example, variant 3 shows stronger conservation of bulky side-chains at the two positions (Trp and His); or the presence of short side-chains (Gly, Ala) at position 33 in variant 4 allows parts of CDR-H3 to fold on top of CDR-H1. Some other subtle structural differences include an opening of the side-chains at position 31 and 32 (variant 2) through a backbone motion at positions 30-32 and a movement in Phe-27 bringing it closer to CDR-H3 (variant 3) through a backbone motion at position 27-30.

The five variants of CDR-H2 are mapped to the canonical structure H2-10-1 (**Supplementary Table 2**) and differ in the rotation at position 52A affecting the Cβ orientation of the side-chain (**Supplementary Figure 3B**). The variants can be ordered in terms of side-chain exposure and contribution to P1 occupancy as follows: 5 > 4 > 1 = 2 = 3. (**Supplementary Figure 6**). The variant 4 (H2-10-1.1.4) is slightly less conserved at positions 51 (Ile) and 57 (Thr) and shows some presence of hydrophobic side-chains at position 57 (Ile/Leu) interfering with CDR-H2. The variant 5 (H2-10-1.1.5) has the highest exposure of side-chain at position 52A and it is the only variant that does not maintain conservation of Pro-52A. All variants display mild compensatory structural changes from CDR-H1 of 2-3% occupancy of the P1 pocket.

2.2 Species-related preferences in topologies

The previous sections shed some light on the nature of residues at some key positions that may guide sequence preferences in the CDR and the framework. Structural databases only englobe a tiny fraction of the whole antibody sequence repertoire. To obtain a more accurate and global view of antibody sequence diversity, genomic databases were leveraged. Unique genomic sequences of antibodies with CDR-H1–CDR-H2 configuration 13-10 were collected and analyzed. Sequence conservation profiles for the different antibody classes were compared (**Supplementary Figure 9**). Overall, there is a fairly good agreement between sequence conservation of the structural and genomic data all pointing out that the PDB sample of our dataset was representative.

From the profiles, one can deduce some preferences in the canonical structures and topologies given the structurally important residues described in this study. One striking observation of sdAbs sequences (both from llama and alpaca) as opposed to IGHV3 or IGHV1 mAbs is their overall higher variability of sequence within the framework (including the support set of residues) and the CDR (including the positions highlighted as structurally important). In IGHV3 and IGHV1 mAbs, we observed a strong consensus for H1-13-1.1 given the high propensity for Phe and Tyr at position 27, along with position 29 being almost exclusively observed as Phe. In sdAbs, Phe is still conserved (H1-13-1.1) at position 29 but position 27 shows more variability to short hydrophobic side-chains (H1-13-1.2) and polar side-chains (H1-13-5.1). The sdAbs from alpaca appear to have a higher population of the H1-13-5.1 topology than for llama given the increased propensity for Ile at position 28, with an increased propensity for polar side-chains at position 27. Surprisingly, bulky side-chains are not abundant for alpaca at position 52A, which would normally be required to fill the P1 and P2 pockets. On the other hand, sdAbs from llama are more dominant in H1-13-1.1 as perceived by its higher conservation of Thr-28, Phe-29 and Tyr-32.

An inverse correlation is observed at positions 54 and 55 underlining the population distribution for H2-10-1 (Gly-55-dominant such as in IGHV1 and sdAbs from alpaca) and H2-10-2 (Gly-54-dominant such as in IGHV3). The sdAbs from llama are more evenly distributed with a bias for H2-10-2. Arg-71 is conserved throughout species and antibody classes but more heavily so in those dominant for H2-10-2 requiring Arg-71 for P1 packing. There is a strong relation across species between the conservation patterns for residues at CDR-H1 position 29 and CDR-H2 positions 54 and 55, highlighting some preferences in the combination of CDR-H1 and CDR-H2 sequences. For sdAbs and related IGHV3 antibodies, the cases that have an increased propensity for Phe-29 are associated with an increased propensity for Gly-54, whereas the cases with a reduced propensity for Phe-29 are associated with an increased propensity for Gly-55. For IGHV1, the higher propensity for Tyr-27 favours Gly-55.

3 Supplementary References

1. Altschul SF, Gish W, Miller W, Myers EW, Lipman DJ. Basic local alignment search tool. *J Mol Biol* (1990) **215**:403–410. doi:10.1016/S0022-2836(05)80360-2
2. Peng HP, Lee KH, Jian JW, Yang AS. Origins of specificity and affinity in antibody-protein interactions. *Proc Natl Acad Sci U S A* (2014) **111**: doi:10.1073/pnas.1401131111

4 Supplementary Figures and Tables

4.1 Supplementary Tables

Supplementary Table 1. Properties of CDR-H1 and CDR-H2 clusters for IGHV3 antibodies

	Centroid structure	Topological variant[a]	Sequence diversity[b]		RMSF[c]		Count[d]			
			Loop	Paired	Average	Max	N	R71	A94	K_{base}
CDR-H1	6xul[C]	H1-13-1.1.1	4.5 ± 1.7	6.9 ± 1.6	0.31	0.55	132 (42)	125 (95)	0 (0)	127 (96)
	6vjt[H]	H1-13-1.1.2	4.0 ± 1.8	6.4 ± 1.9	0.28	0.52	78 (25)	78 (100)	0 (0)	68 (87)
	6xsw[D]	H1-13-1.1.3	5.4 ± 1.3	7.5 ± 1.4	0.37	0.6	20 (6)	8 (40)	0 (0)	19 (95)
	5tdo[B]	H1-13-1.2.1	4.4 ± 1.7	4.9 ± 1.7	0.41	0.85	44 (14)	1 (2)	0 (0)	44 (100)
	3inu[H]	H1-13-1.2.2	5.8 ± 1.6	7.4 ± 1.6	0.53	0.9	22 (7)	14 (64)	0 (0)	20 (91)
CDR-H2	5uea[A]	H2-10-1.1.1	5.9 ± 1.5	5.9 ± 1.9	0.36	0.98	49 (16)	4 (8)	0 (0)	49 (100)
	4xgz[E]	H2-10-1.1.2	4.8 ± 2.0	4.3 ± 2.1	0.33	0.67	23 (7)	0 (0)	0 (0)	23 (100)
	4zyk[H]	H2-10-2.1.1	6.2 ± 1.9	4.4 ± 1.9	0.27	0.48	101 (32)	101 (100)	0 (0)	92 (91)
	6pzh[H]	H2-10-2.1.2	6.7 ± 1.8	4.4 ± 1.9	0.25	0.39	61 (19)	61 (100)	0 (0)	55 (90)
	4ydv[B]	H2-10-2.1.3	6.6 ± 1.5	4.9 ± 2.2	0.39	0.87	33 (10)	31 (94)	0 (0)	32 (97)

[a] The topological variant of the cluster as referenced by the predicted canonical structure according to PyIgClassify suffixed with the topology ID followed by variant ID. The canonical structure was obtained by finding the most frequent canonical structure among representatives of the cluster. The clusters were ordered by topologies and variants in terms of structure representation.
[b] Sequence diversity is quantified through a pairwise sequence comparison within representatives of a given cluster using a naive Levenshtein distance for the canonical loop of the respective cluster and the paired canonical loop (CDR-H2 for CDR-H1 clusters and CDR-H1 for CDR-H2 clusters).
[c] RMSF as calculated for B-factors of centroids.
[d] Count in number of representatives within the cluster (N), having Arg at position 71 (R71), having Ala at position 94 (A94) and adopting a kinked CDR-H3 base geometry (K_{base}). The percentages shown in parenthesis were calculated out of the total number antibody entries in the case of the N column, or out of the number of cluster representatives (N) in the case of the other columns.

Supplementary Table 2. Properties of CDR-H1 and CDR-H2 clusters for IGHV1 antibodies

	Centroid structure	Topological variant[a]	Sequence diversity[b]		RMSF[c]		Count[d]			
			Loop	Paired	Average	Max	N	R71	A94	K_{base}
CDR-H1	1oaq[H]	H1-13-1.1.1	4.5 ± 1.6	6.3 ± 1.4	0.29	0.49	195 (37)	39 (20)	1 (1)	175 (90)
	5myx[B]	H1-13-1.1.2	4.7 ± 1.7	6.1 ± 1.5	0.33	0.8	100 (19)	17 (17)	0 (0)	86 (86)
	4uik[H]	H1-13-1.1.3	4.5 ± 1.9	5.9 ± 1.5	0.33	0.79	70 (13)	11 (16)	1 (1)	57 (81)
	5k9q[S]	H1-13-1.1.4	5.1 ± 1.7	5.4 ± 1.7	0.37	0.84	33 (6)	7 (21)	3 (9)	31 (94)
CDR-H2	6sxi[A]	H2-10-1.1.1	6.2 ± 1.4	4.9 ± 1.8	0.24	0.41	169 (32)	28 (17)	3 (2)	146 (86)
	3gnm[H]	H2-10-1.1.2	6.1 ± 1.4	4.5 ± 1.7	0.26	0.46	97 (19)	7 (7)	1 (1)	82 (85)
	6w5d[H]	H2-10-1.1.3	5.9 ± 2.0	6.2 ± 1.8	0.35	0.6	45 (9)	1 (2)	0 (0)	44 (98)
	5tqa[A]	H2-10-1.2.1	5.9 ± 1.4	5.5 ± 1.7	0.3	0.57	65 (12)	19 (29)	0 (0)	59 (91)
	5otj[B]	H2-10-1.2.2	5.6 ± 1.6	5.1 ± 1.8	0.32	0.7	40 (8)	17 (43)	1 (3)	34 (85)

[a] The topological variant of the cluster as referenced by the predicted canonical structure according to PyIgClassify suffixed with the topology ID followed by variant ID. The canonical structure was obtained by finding the most frequent canonical structure among representatives of the cluster. The clusters were ordered by topologies and variants in terms of structure representation.
[b] Sequence diversity is quantified through a pairwise sequence comparison within representatives of a given cluster using a naive Levenshtein distance for the canonical loop of the respective cluster and the paired canonical loop (CDR-H2 for CDR-H1 clusters and CDR-H1 for CDR-H2 clusters).
[c] RMSF as calculated for B-factors of centroids.
[d] Count in number of representatives within the cluster (N), having Arg at position 71 (R71), having Ala at position 94 (A94) and adopting a kinked CDR-H3 base geometry (K_{base}). The percentages shown in parenthesis were calculated out of the total number antibody entries in the case of the N column, or out of the number of cluster representatives (N) in the case of the other columns.

4.2 Supplementary Figures

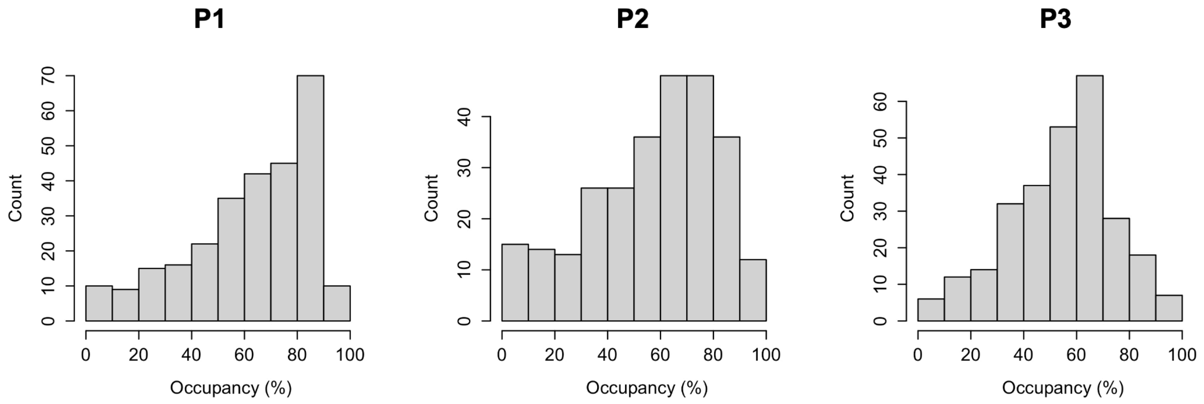

Supplementary Figure 1. Percentage of occupancy of the pockets P1, P2 and P3 for single-domain antibody entries. The pockets can be occupied from a combination of atoms of the CDR-H1, CDR-H2, CDR-H3, FR3 and the N-terminus.

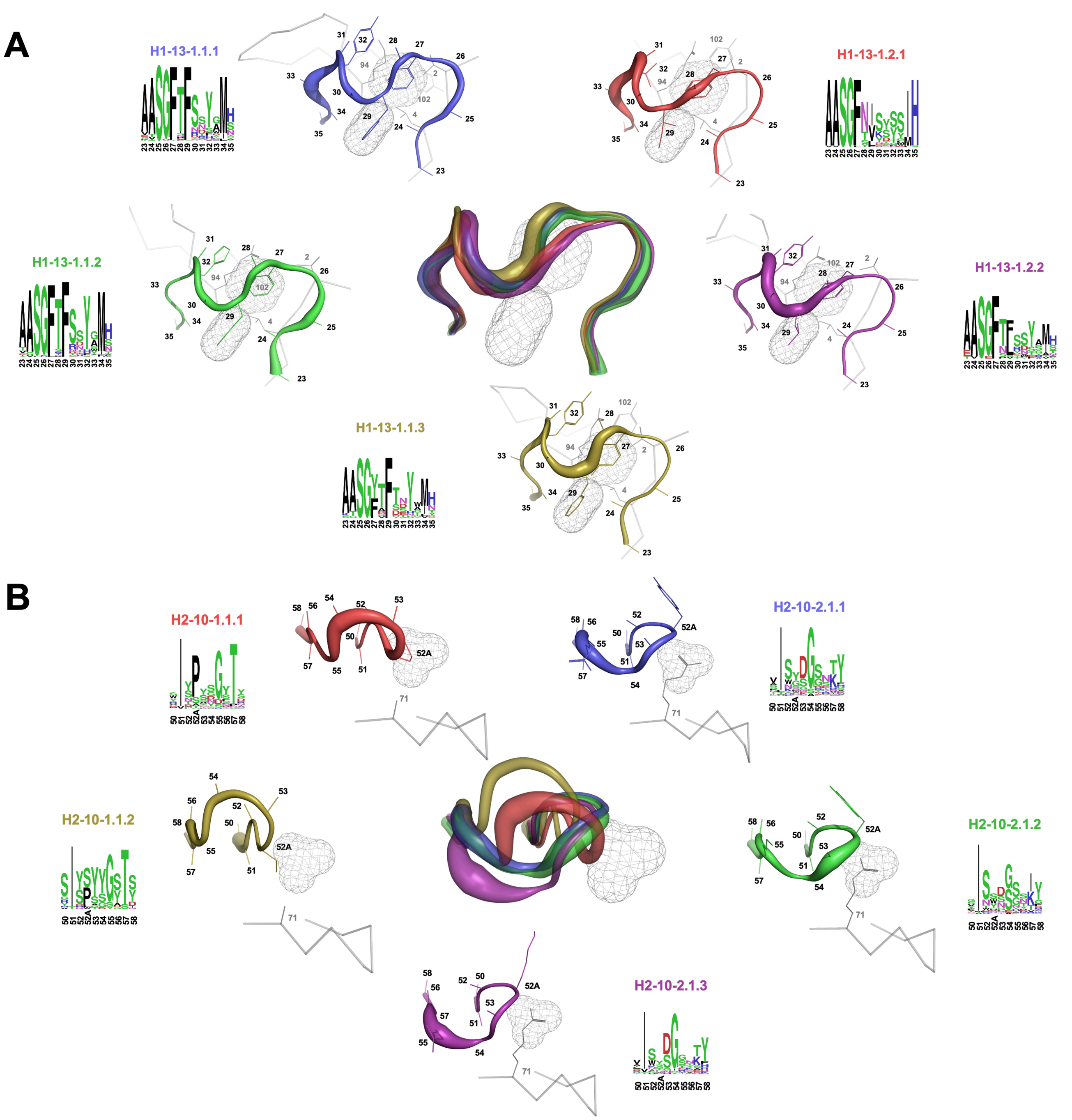

Supplementary Figure 2. Rendering of the major topological variants for CDR-H1 (**A**) and CDR-H2 (**B**) for IGHV3 antibodies. CDR-H1 is displayed in the presence of pockets P2 and P3, and CDR-H2 in the presence of pocket P1 and residue at FR3 position 71. A superposition view depicts structural differences between the variants. The variants are uniquely colored and displayed individually as sausage views around the centroid structure (see **Supplementary Table 1**). The centroid structures were superimposed based on the set of backbone framework atoms. The thickness of the sausage is indicative of the structural fluctuations among cluster members. Each variant is shown with its associated sequence logo with colors denoting the nature of the amino acids.

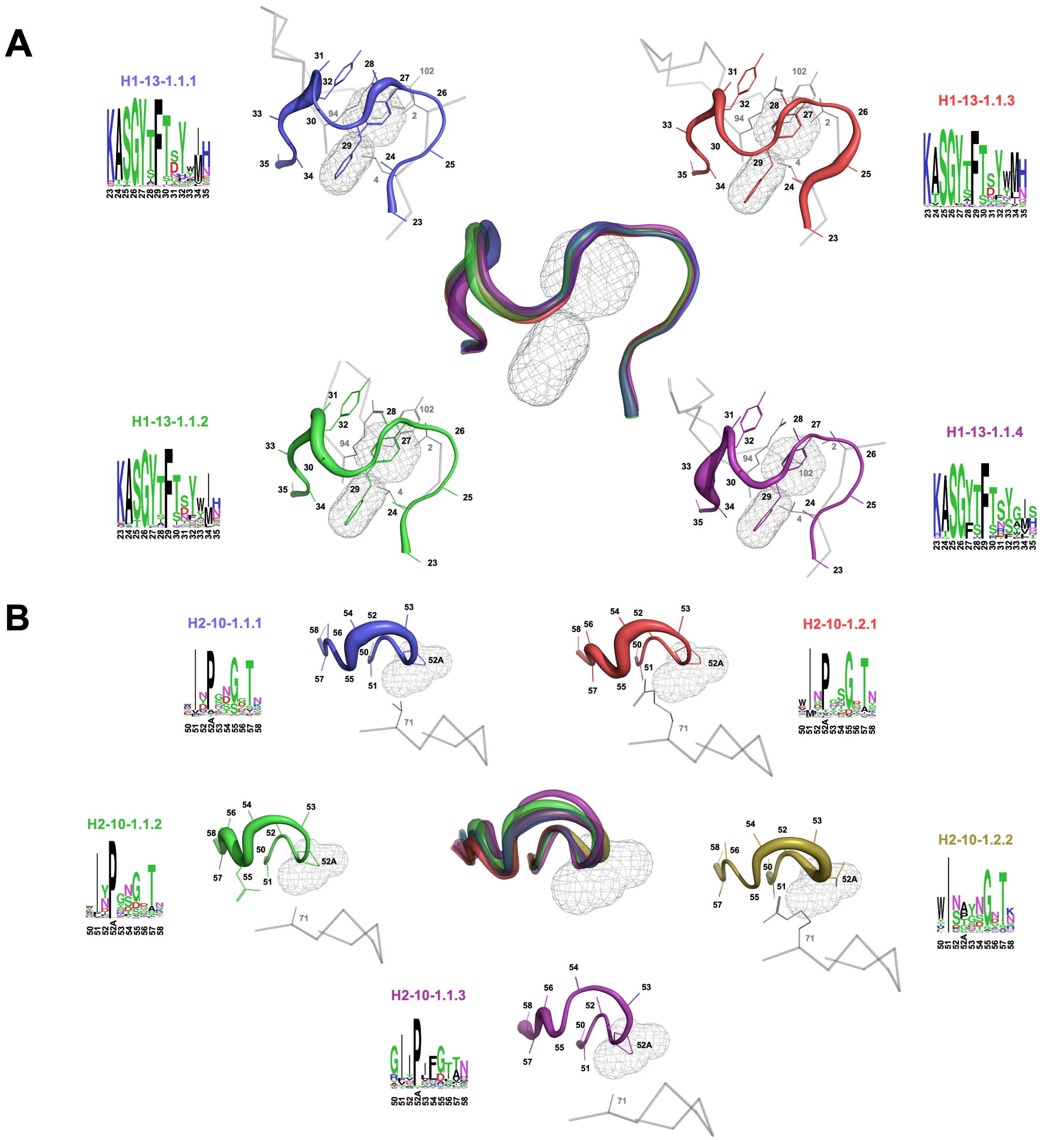

Supplementary Figure 3. Rendering of the major topological variants for CDR-H1 (**A**) and CDR-H2 (**B**) for IGHV1 antibodies. CDR-H1 is displayed in the presence of pockets P2 and P3, and CDR-H2 in the presence of pocket P1 and residue at FR3 position 71. A superposition view depicts structural differences between the variants. The variants are uniquely colored and displayed individually as sausage views around the centroid structure (see **Supplementary Table 2**). The centroid structures were superimposed based on the set of backbone framework atoms. The thickness of the sausage is indicative of the structural fluctuations among cluster members. Each variant is shown with its associated sequence logo with colors denoting the nature of the amino acids.

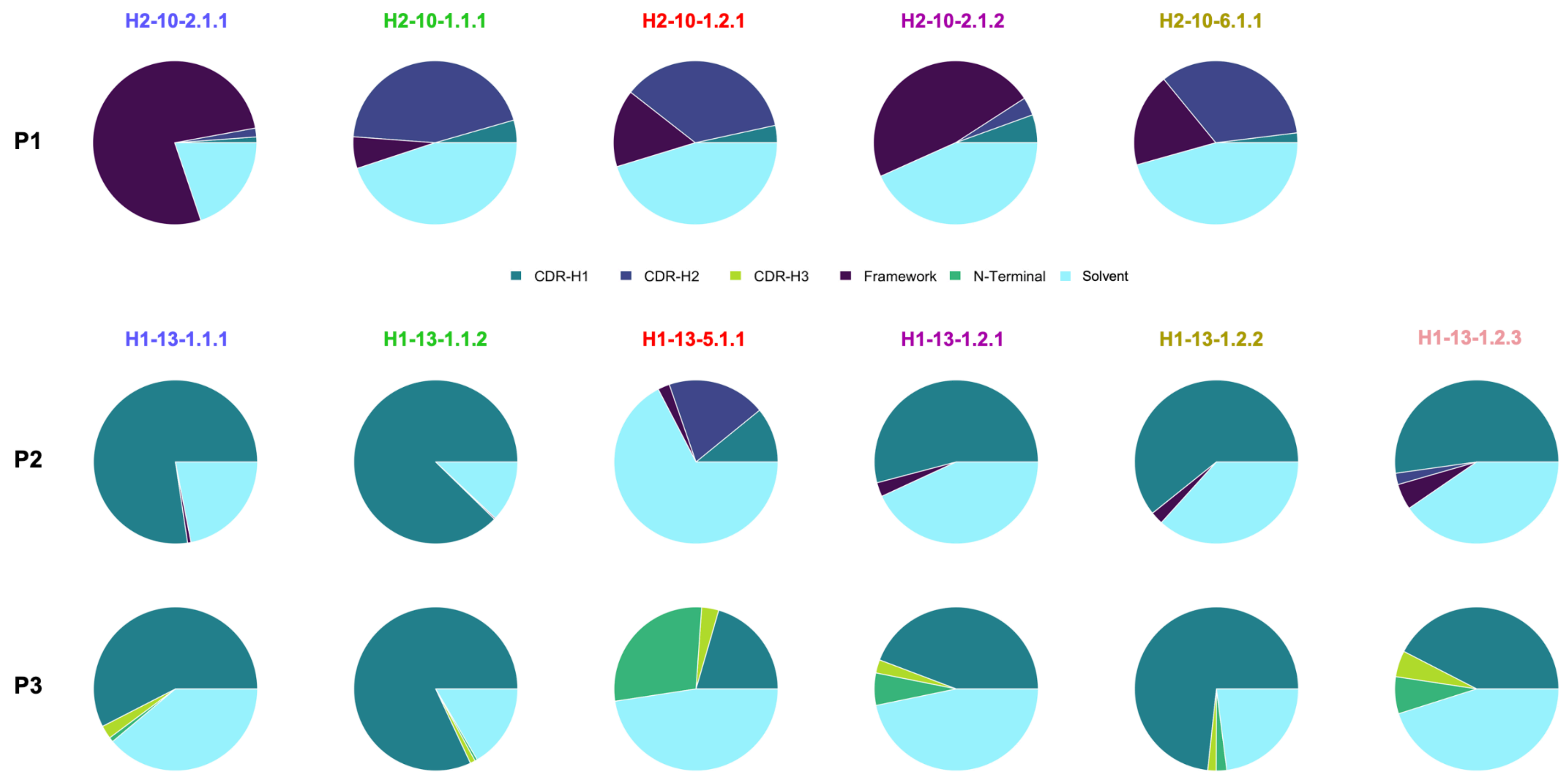

Supplementary Figure 4. Fraction of occupancy of various regions of single-domain antibodies (H1: CDR-H1; H2: CDR-H2; H3; CDR-H3; FR3: a segment of FR3; NT; a segment of the N-terminal) contained within the volume of the pockets P1, P2 and P3. The unoccupied volume of the pockets is declared as being occupied by the solvent. The fractions calculated are averages from the set of cluster (or variant) representatives. Only fractions for the major variants are displayed.

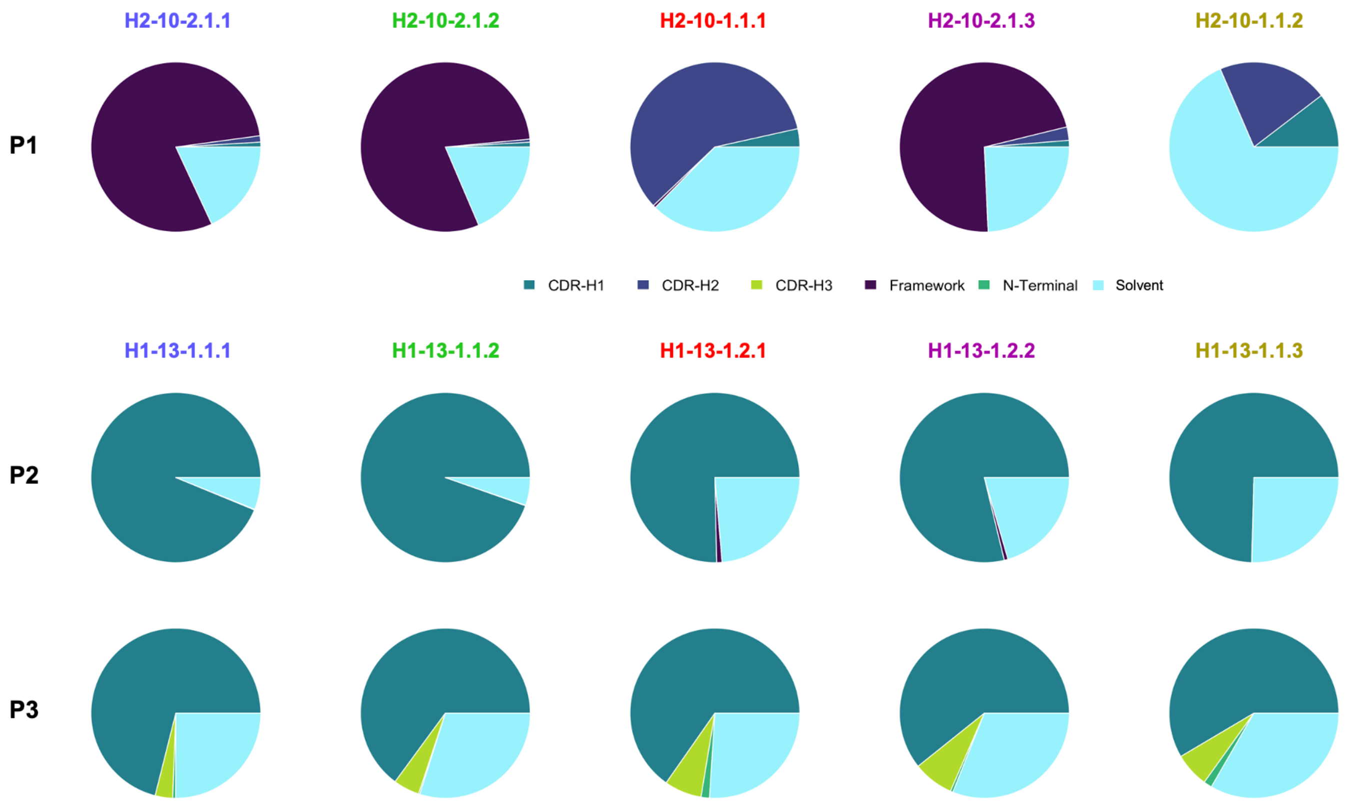

Supplementary Figure 5. Fraction of occupancy of various regions of IGHV3 antibodies (H1: CDR-H1; H2: CDR-H2; H3; CDR-H3; FR3: a segment of FR3; NT; a segment of the N-terminal) contained within the volume of the pockets P1, P2 and P3. The unoccupied volume of the pockets is declared as being occupied by the solvent. The fractions calculated are averages from the set of cluster (or variant) representatives. Only fractions for the major variants are displayed.

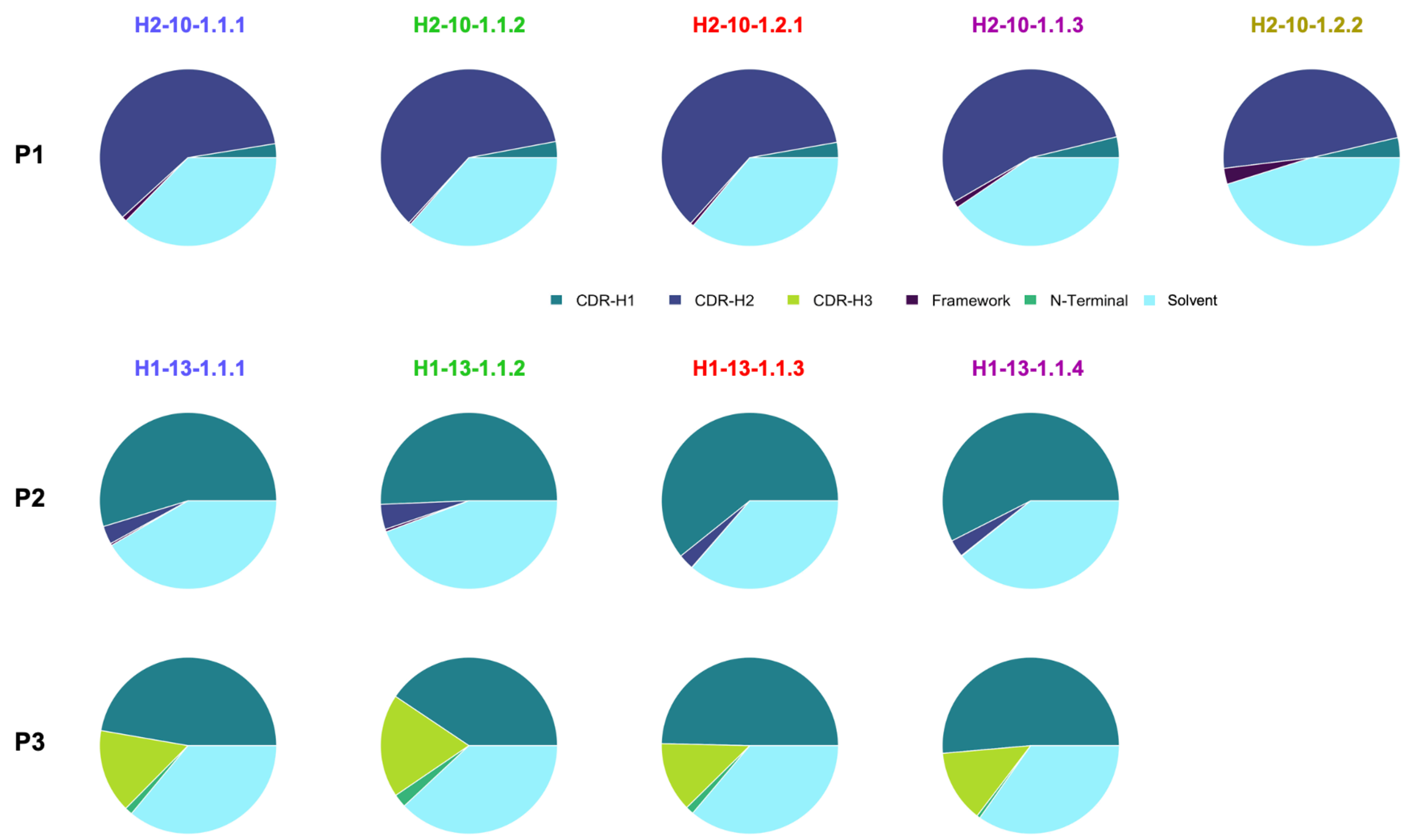

Supplementary Figure 6. Fraction of occupancy of various regions of IGHV1 antibodies (H1: CDR-H1; H2: CDR-H2; H3; CDR-H3; FR3: a segment of FR3; NT; a segment of the N-terminal) contained within the volume of the pockets P1, P2 and P3. The unoccupied volume of the pockets is declared as being occupied by the solvent. The fractions calculated are averages from the set of cluster (or variant) representatives. Only fractions for the major variants are displayed.

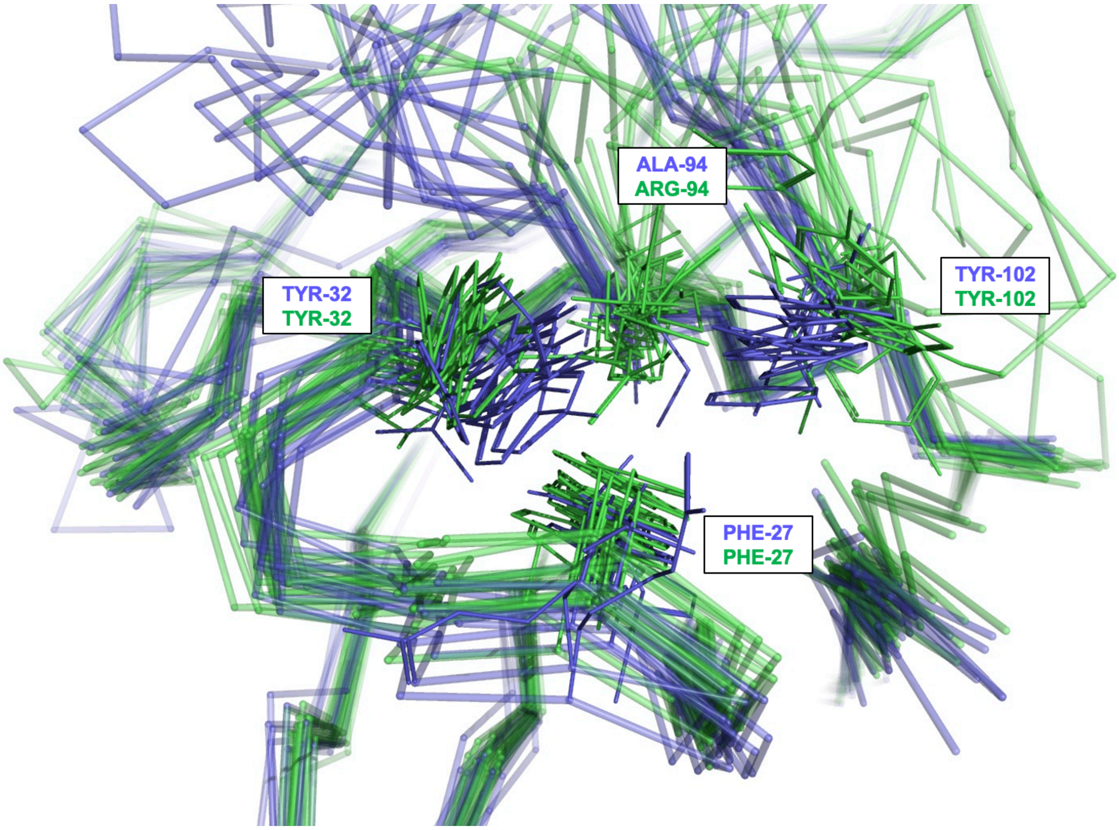

Supplementary Figure 7. Rendering of population shift upon residue changes at structurally determining positions. The presence of Ala at CDR-H3 position 94 (H1-13-1.1.1 in blue) leads to a closed form of CDR-H1 in opposition to Lys/Arg-94 (H1-13-1.1.2 in green) that leads to an open form as observed in variants of sdAbs.

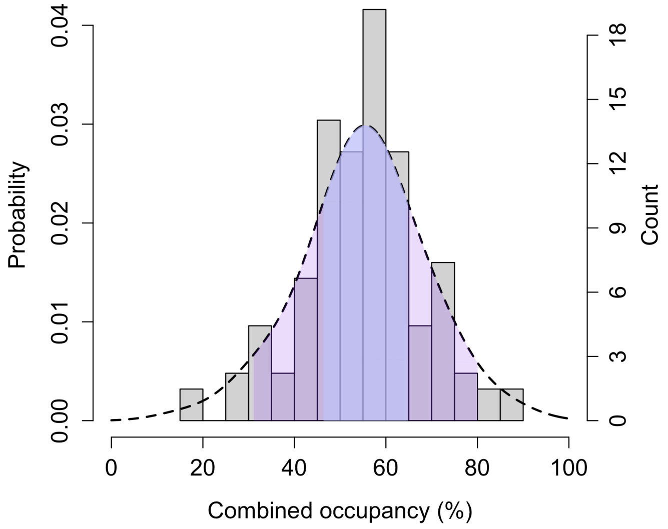

Supplementary Figure 8. Probability distribution for the combined occupancy (P1+P2+P3) for all combinations of CDR-H1 variants against CDR-H2 variants (including both minor and major variants). The dashed line is a smoothed density curve obtained from the histogram. The blue and purple shaded regions encompass 50% and 90% of the data points.

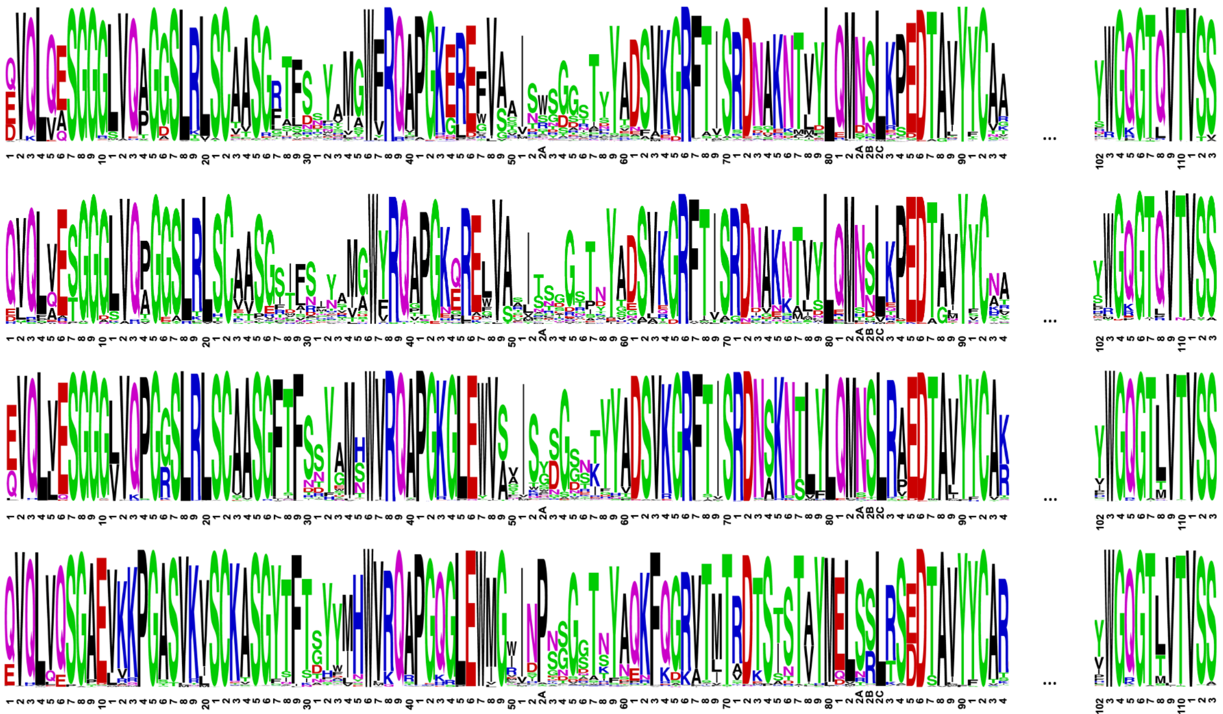

Supplementary Figure 9. Sequence conservation profiles in sdAbs (llama: first row; alpaca: second row) and VH (human IGHV3: third row and human IGHV1: fourth row) from NCBI sequence database. Only unique sequences in CDR-H1-H2 were retained. The number of unique BLAST hits are 474, 87, 587 and 399 respectively.
